# Miniaturized Electronic Noses for Food Quality and Safety: Applications in Lipid Oxidation Monitoring

**DOI:** 10.3390/molecules31193424

**Published:** 2026-09-26

**Authors:** Maxence Floch, Aynur Gunenc, Farah Hosseinian

**Affiliations:** 1 Food Science, Chemistry Department, Carleton University, Ottawa, ON K1S 5B6, Canada; maxence.floch@agrosupdijon.fr (M.F.); aynurgunenc@cunet.carleton.ca (A.G.); 2 Institut Agro Dijon, National Institute of Higher Education for Agriculture, Food and Environmental Sciences, 21079 Dijon, France; 3 Institute of Biochemistry, Carleton University, Ottawa, ON K1S 5B6, Canada

**Keywords:** e-nose, lipid oxidation, real-time measurement, food-quality monitoring, volatile compounds, sensor arrays

## Abstract

Lipid oxidation is a major degradation process in lipid-rich food systems, producing volatile organic compounds (VOCs) associated with rancidity, off-flavours, and reduced shelf life. Although conventional techniques such as gas chromatography–mass spectrometry (GC-MS) provide reliable identification and quantification of oxidation products, their laboratory-based operation and complex sample preparation limit their suitability for rapid, real-time monitoring. This review examines recent advances in miniaturized and portable electronic-nose (e-nose) systems for monitoring lipid oxidation and food quality through volatile fingerprint analysis. Unlike chromatographic techniques, e-noses generate characteristic response patterns using sensor arrays coupled with pattern recognition and machine learning algorithms rather than directly identifying individual compounds. The relationship between oxidation-derived volatiles, including aldehydes, ketones, alcohols, and hydrocarbons, and e-nose responses are discussed across various food matrices. Metal oxide semiconductor (MOS) sensors remain the most widely investigated technology because of their robustness, scalability, and portability, while electrochemical, conducting polymer, quartz crystal microbalance (QCM), optical, field-effect transistor (FET), and surface acoustic wave (SAW) sensors are also reviewed. Recent developments in chemometric, machine learning, and deep learning approaches for classification, prediction, and sensor-drift compensation are highlighted, alongside applications in edible oils, meat, dairy products, fermented foods, beverages, coffee, and cocoa. Despite considerable progress, sensor drift, humidity interference, calibration transfer, reproducibility, and limited industrial validations remain important challenges. Future research should prioritize sensor stability, standardized validation protocols, and integration with smart-food-monitoring platforms for reliable real-time quality assessment and shelf-life applications.

## 1. Introduction

Lipid oxidation is one of the main causes of quality deterioration in fat-containing foods, affecting sensory properties, nutritional quality and shelf life. This process involves free-radical reactions that promote the degradation of unsaturated fatty acids and the formation of volatile organic compounds (VOCs), including aldehydes [1], ketones [2], hydrocarbons [3], and alcohols [4]. Primary oxidation products, particularly lipid hydroperoxides, subsequently decompose into secondary volatile compounds that contribute to rancidity, colour changes, and undesirable odours. Among these compounds, aldehydes such as hexanal and nonanal are important indicators of oxidative deterioration due to their strong association with rancid flavours. Consequently, oxidation-derived VOCs provide valuable information for monitoring lipid deterioration and are important targets for volatile fingerprint-based technologies [5].

Reliable monitoring of lipid oxidation is essential for food-quality control during production, storage, transportation, and retail. Conventional analytical techniques, including high-performance liquid chromatography (HPLC) and gas chromatography–mass spectrometry (GC-MS), provide accurate characterization and quantification of oxidation products but are often associated with high cost, complex sample preparation, laboratory-based operation, and limited suitability for continuous monitoring [6]. These limitations have encouraged the development of rapid, non-destructive, and portable technologies for food-quality assessment. Electronic-nose (e-nose) systems are among the technologies that have attracted increasing attention. An e-nose typically combines an array of chemical complex mixtures of VOCs through collective sensor responses. Unlike the chromatographic techniques, e-noses generally do not identify individual compounds directly but generate characteristic response patterns that can be used for simple discrimination and classification. Therefore, e-nose measurements can provide rapid volatile fingerprinting, while complementary analytical methods remain important when compound-specific or quantitative information is required.

The miniaturization of e-nose systems has further increased their potential for on-site and real-time food monitoring. Miniaturized platforms can integrate compact sensor arrays, low-power electronics, data processing, and wireless communication into portable devices, facilitating measurements in production, storage, transportation, and retail environments. Unlike conventional laboratory e-noses, which are generally benchtop instruments equipped with complex sampling units and high-power electronics, the mini electronic noses incorporate compact detection modules, low-power microcontrollers, wireless communication capabilities, and integrated data processing into lightweight battery-powered platforms. Their small size—5 cm long by 2 cm wide—and energy consumption make them suitable for real-time monitoring in food production, storage, transport and retail environments [7]. Mini e-noses have consequently emerged as promising tools for rapid and non-destructive assessment of food quality [8]. However, the interpretation of sensor responses in relation to the underlying chemical processes remains an important challenge. Establishing the relationship between oxidation-derived VOC profiles, conventional oxidation indicators, and sensor responses is essential to improving the reliability and practical value of these systems.

Mini e-noses offer a promising approach for the rapid and non-destructive assessment of volatile fingerprints associated with lipid oxidation [9], meats [10], or dairy products [11], and previous studies have demonstrated their potential to classify different oxidation levels and predict product shelf life. However, previous reviews have mainly examined e-noses in broad food-quality monitoring, and most have focused on broad applications such as freshness assessment, microbial spoilage detection, authentication, fermentation, adulteration, or ripening. Comparatively, less attention has been given to miniaturized e-nose systems, specifically for monitoring lipid oxidation, the evolution of oxidation-related VOC fingerprints, and their relationship with conventional oxidation indicators.

Despite the increasing development of mini e-noses systems, several research gaps remain before these technologies can be reliably implemented for real-time monitoring of lipid oxidation in food products. First, the relationship between the volatile fingerprints detected by the mini e-noses and the actual progression of lipid oxidation remains insufficiently established. Although electronic noses can detect changes in VOC profiles, the specific contribution of individual oxidation products and their relationship to established oxidation markers such as TBARS, aldehydes and other secondary oxidation products are not always clearly characterized. This limits the ability of mini e-noses to provide quantitative and chemically interpretable information rather than simply distinguishing between fresh and oxidized samples.

Furthermore, most studies were conducted under controlled laboratory conditions, while real-time monitoring of food involves substantial variations in temperature, relative humidity, oxygen availability, composition of the samples and headspace conditions. These factors can strongly influence sensor responses and could reduce the transferability of models developed under controlled conditions to real food environments. Sensor drift and changes in sensor sensitivity over time also remain significant challenges, especially for long-term monitoring and repeated measurements.

The performance of mini e-nose systems is also influenced by the characteristics of the sensor technologies used. Metal oxide semiconductor (MOS) sensors remain the most widely investigated platform due to their robustness, scalability, and compatibility with mini devices. However, alternative sensing technologies, including electrochemical, conducting polymer, quartz crystal microbalance (QCM), optical, field-effect transistor (FET), and surface acoustic wave (SAW) sensors, have also been investigated. These technologies differ in terms of sensitivity, selectivity, response time, operating conditions, power consumption, and long-term stability. Therefore, comparing the analytical performance, advantages, and limitations of different sensor platforms is important for evaluating their suitability for real-time food-monitoring applications.

The aim of this review is to critically examine recent advances in miniaturized e-nose technologies for real-time monitoring of lipid oxidation in food systems. Emphasis is placed on sensor technologies, oxidation-related VOC fingerprints, machine learning and chemometric approaches, and the relationship between e-nose responses and conventional analytical methods such as GC-MS and peroxide value measurements. The review considers applications across different food matrices, including oils, meat, dairy products, and other food systems, and evaluates the potential of mini e-noses for rapid assessment and prediction of oxidation and shelf life. Current limitations, including sensor drift, environmental variability, humidity effects, calibration transfer, reproducibility, and limited industrial validation, are also critically examined.

### Comparison with Previous Reviews and Novelty of the Present Review

Unlike previous reviews that have broadly discussed electronic noses (e-noses) for food-quality assessment, the present review focuses specifically on miniaturized e-nose systems, with particular emphasis on their application to monitoring lipid oxidation in food matrices. At the same time, relevant applications of mini e-noses for other important food-quality and safety aspects, including microbial spoilage, fermentation, adulteration, ripening, and physical damage, are also considered. Attention is given to the detection of volatile organic compounds (VOCs) generated during lipid oxidation and to the evolution of their volatile fingerprints across different food matrices, including oils, meat, and dairy products. In addition, this review goes beyond the general description of e-nose technologies provided in previous reviews by examining the relationship between e-nose responses and conventional reference methods used to assess lipid oxidation, including GC–MS and TBARS. The present review also provides a critical assessment of the practical challenges associated with miniaturized e-nose deployment, including sensor drift, sensitivity, humidity, temperature, and other environmental effects. By integrating these aspects, the present review provides a more focused evaluation of the specific potential and current limitations of miniaturized e-noses as rapid and non-destructive tools for early monitoring of lipid oxidation and food quality. Furthermore, it identifies key technological gaps, including sensor stability, calibration transfer, environmental robustness, and validation under realistic conditions, that need to be addressed to facilitate the wider application of miniaturized e-noses in real-world and industrial food-monitoring environments.

## 2. Lipid Oxidation

Lipid oxidation is a series of undesirable chemical reactions [12] that cause the degradation of fatty acids found in foods such as cheese [13], meat [14], and cooking oils [15]. It is recognized as one of the primary causes of food spoilage [16] and quality deterioration [17], leading to rancidity and the formation of off-flavour compounds [18].

Multiple **intrinsic elements** have an impact on lipid oxidation within food systems. This includes not only the analysis of fatty acid composition and natural antioxidants [19], but also a series of pro-oxidative and structural factors that determine oxidation dynamics and processes. For starters, the fatty acid profile, especially the level of unsaturation, is crucial. Polyunsaturated fatty acids (PUFAs) are very sensitive to oxidation due to the presence of multiple double bonds [20]. Second, oxidation is greatly accelerated by the presence of pro-oxidants, such as transition metal ions such as iron [21] and copper [22] and proteins such as myoglobin and haemoglobin [23]. Moreover, the number of phospholipids can have an impact on oxidation [24] due to their rich content of polyunsaturated fatty acids and their amphiphilic character [25]. The activity of enzymes is also crucial, especially enzymes such as lipoxygenases [26]. They catalyse the oxygenation of unsaturated fatty acids, leading to the production of hydroperoxides and secondary oxidation products. In addition, the physical composition of the food matrix such as bulk oils compared to emulsions significantly influences the oxidation process [27]. In emulsified systems, oxidation tends to accelerate due to the large interfacial surface between lipids and the aqueous phase, where pro-oxidants are more easily accessible [28].

**External factors** significantly influence the kinetics and oxidation pathways of lipids in food systems [29]. These factors also influence the composition and concentration of oxidation-derived VOCs, thereby affecting the volatile fingerprints detected by e-nose systems. Temperature is one of the most critical parameters, as the increase in temperature accelerates reaction [30] rates by promoting the formation of radicals and the decomposition of lipid hydroperoxides [31], thus favouring chain propagation reactions [32]. Exposure to light induces photo-oxidation by the generation of singlet oxygen [33], which reacts rapidly with unsaturated fatty acids and leads to the formation of hydroperoxides without the need for radical initiation [34]. The partial pressure of oxygen directly affects the availability of molecular oxygen necessary for lipid peroxidation [35]. Water activity plays a non-linear role in lipid oxidation. At low moisture levels, oxidation is often enhanced due to the increased mobility of oxygen and pro-oxidants at lipid interfaces [36]. Finally, processing and storage conditions strongly modulate oxidation [23] pathways by modifying the lipid structure [37], increasing surface exposure.

Lipid oxidation is a complex chain reaction that deteriorates the quality of the product through chemical and sensory changes, as illustrated in Figure 1. Lipid peroxidation begins when an oxidizing species (hydroxyl radical, peroxyl radical…) removes an allylic hydrogen from a polyunsaturated fatty acid, generating a carbon-cantered lipid radical (R•) [38]. This radical rearranges by electronic delocalization to form a conjugated diene, a more stable structure that reacts very quickly with molecular oxygen to give a peroxyl radical (RO2•). The latter, in turn, extracts hydrogen from a neighbouring fatty acid, forming a lipid hydroperoxide and regenerating a new radical R•: this is the propagation phase, which maintains the chain reaction and can affect many successive lipid molecules [39]. This step is also catalysed by transition metals (Fe^2+^/Fe^3+^), which decompose the hydroperoxides into additional alkoxy or peroxyl radicals (Fenton reaction), further amplifying the process when prooxidants such as the hydroxyl radical rip off allylic hydrogen to form the carbon-cantered lipid radical, which then stabilizes by molecular rearrangement into a conjugated diene [40]. The peroxyl radical formed then reacts rapidly with a new fatty acid to propagate the radical chain.

The alkoxylate and peroxyl radicals resulting from primary oxidation then evolve in two distinct ways. The first is degradation by cleavage: the carbon chain fragments and releases reactive low-molecular-weight aldehydes, in particular malondialdehyde (MDA) and 4-hydroxynonenal (4-HNE), recognized as the most studied end products of lipid peroxidation and as biological markers of oxidative stress. The second route, specific to fatty acids such as arachidonic acid, involves an intramolecular cyclization of the peroxyl radical, which forms a bicyclic endoperoxide; this is then reduced to give isoprostanes, compounds with a structure like prostaglandins (such as 8-iso-PGF2α) but produced non-enzymatically, making them particularly reliable markers of oxidative stress in vivo [41].

The decomposition of lipid hydroperoxides also generates a wide range of volatile compounds, including aldehydes, ketones, alcohols, hydrocarbons, furans, and organic acids, which are responsible for the characteristic off-flavours associated with lipid oxidation. Among these, aldehydes such as hexanal, pentanal, and nonanal are commonly used as indicators of oxidative deterioration because they are closely associated with sensory-quality changes in food products. These oxidation-derived VOCs constitute the volatile fingerprints detected by e-nose systems. Although e-noses do not identify individual compounds, changes in the overall VOC profile can be correlated with conventional analytical methods such as GC-MS and TBARS, enabling rapid assessment of lipid oxidation [42].

**Figure 1:**
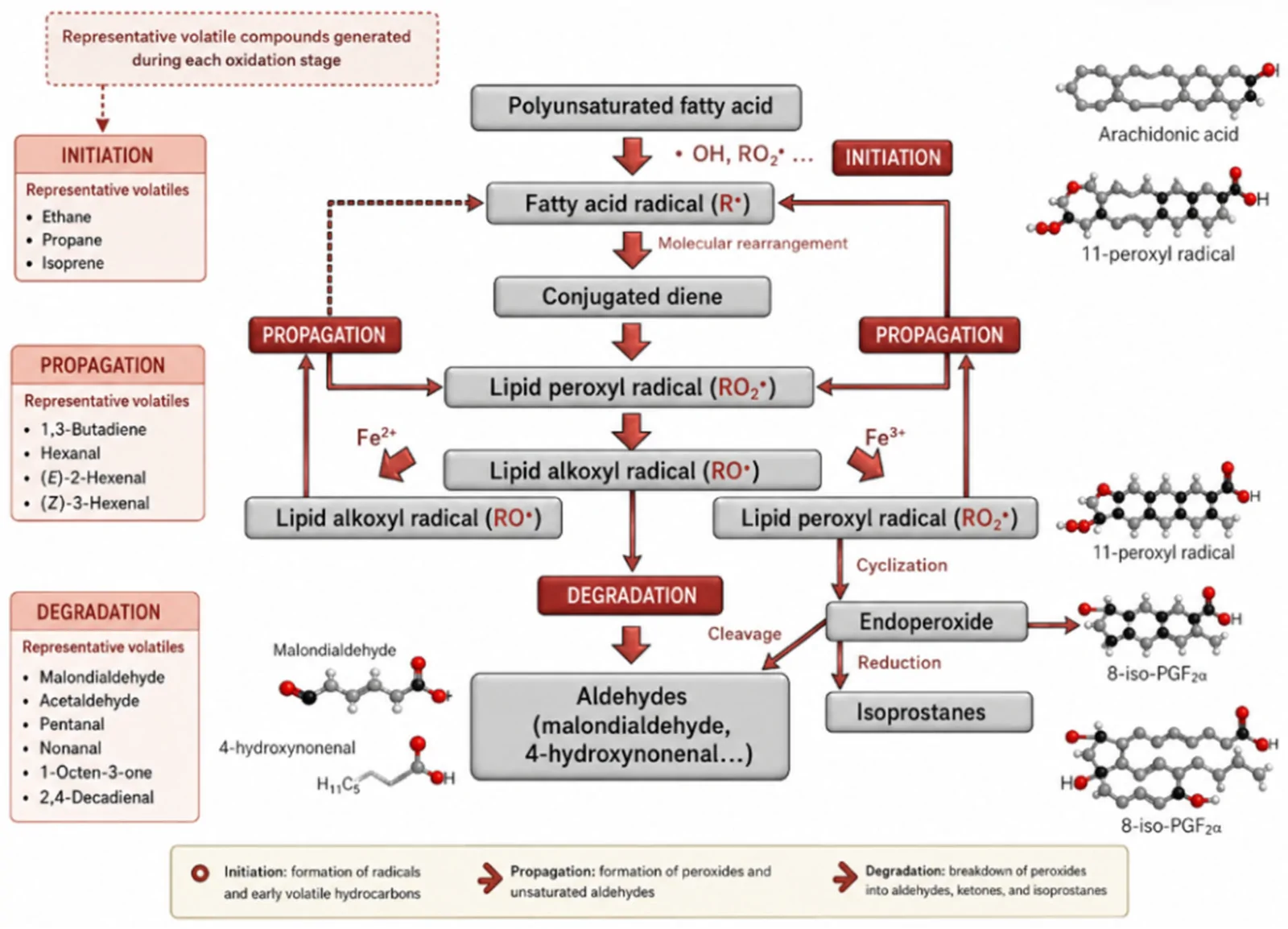
General diagram of lipid oxidation. Adapted from [43] using BioRender (https://www.biorender.com/).

During the final stage of lipid oxidation, chain-linked radical reactions are gradually completed, leading to the creation of a wide range of stable and non-radical compounds. Essentially, these products result from the degradation of lipid hydroperoxides, which are the primary oxidation products generated during the propagation phase. Due to their unstable nature, hydroperoxides undergo a complex cleavage process that produces secondary oxidation elements such as aldehydes, ketones, alcohols, hydrocarbons and esters. In addition, other rearrangement and oxidation reactions can generate non-volatile compounds, such as epoxides and ether oxides. These molecules are no longer radical species, but they contribute significantly to the chemical degradation of lipids; they largely contribute to the creation of unpleasant odours, rancid odours and a general deterioration in quality in the food system. Table 1 summarizes the variety of these oxidation products, whether volatile or not.

Among these compounds, hexanal is widely recognized as the primary marker for the oxidation of linoleic acid (C18:2, ω-6) and is frequently used to monitor oxidative spoilage in vegetable oils, meat, dairy products and nuts. Oxidation of α-linolenic acid (C18:3, ω-3) produces compounds such as propanal, 2,4-heptadienal, 2-pentenal and 1-pentene-3-ol, while oleic acid (C18:1) primarily generates nonanal, octanal and decanal. Consequently, different food matrices exhibit distinct VOC profiles depending on their fatty acid composition, which allows e-nose systems to discriminate oxidation states based on global volatile fingerprints rather than individual compounds [44].

Volatile oxidation products are particularly attractive targets for mini e-nose systems because they provide a direct and non-destructive means of monitoring changes occurring in the headspace above oxidizing food matrices. Unlike conventional lipid oxidation indicators, which generally require sample collection, chemical extraction, reagents, or laboratory-based instrumentation, VOC analysis can be performed directly on the headspace with minimal or no sample preparation. Primary and secondary lipid oxidation reactions generate a complex mixture of volatile compounds, whose concentrations and relative proportions evolve as oxidation progresses. This dynamic volatile fingerprint can therefore provide information on the overall oxidative state of a food matrix rather than relying on a single chemical marker. This characteristic is particularly well suited to e-noses, which are designed to recognize patterns generated by mixtures of VOCs rather than identify individual compounds.

This study focuses specifically on VOCs generated during lipid oxidation, as these compounds represent the primary targets detected by mini e-nose systems for real-time monitoring of food-quality changes [45]. These oxidation-derived VOCs serve as important indicators of oxidative degradation and have been widely investigated for their potential use in predicting food shelf life [46]. By continuously monitoring VOC profiles, mini e-nose systems can support rapid quality assessment and provide valuable information regarding the progression of lipid oxidation across different food matrices [47]. Such real-time monitoring approaches are particularly relevant for food manufacturers, as they can contribute to improved shelf-life prediction and quality control during processing and storage.

## 3. Conventional Analytical Methods

GC-MS is a standard and primary method for VOCs analysis in lipid oxidation [48]. GC-MS and HPLC are laboratory-based analytical techniques commonly used for the characterization of lipid oxidation products, whereas e-noses provide rapid, non-destructive assessment of volatile fingerprints associated with oxidation-related changes. More specifically, GC-MS is the primary analytical technique used for the identification and quantification of VOCs generated during lipid oxidation in foods [49], while HPLC is more commonly applied to non-volatile or derivatized oxidation products [50]. Although conventional analytical methods provide highly reliable qualitative and quantitative information, they remain laboratory-based techniques and are therefore mainly used as reference methods for validating the performance of rapid sensing technologies such as e-noses. Of the conventional methods, HPLC is first discussed below because of its important role in analysing non-volatile lipid oxidation products. Unlike GC-MS, which targets VOCs, HPLC is better suited for compounds that are less volatile and require separation in the liquid phase.

### 3.1. Gas Chromatography–Mass Spectrometry (GC-MS)

Unlike HPLC, which is mainly used for non-volatile oxidation products, GC-MS is the principal analytical technique used for the separation, identification, and quantification of volatile and semi-volatile compounds generated during lipid oxidation in complex food matrices. It combines two complementary techniques: gas chromatography (GC), which separates the compounds, and mass spectrometry (MS), which identifies them.

First, the sample is vaporized and carried by a carrier gas, such as helium, through a chromatographic column. The column contains a stationary phase that interacts with compounds based on their volatility and polarity [51]. These interactions result in retention times that vary depending on the compound’s affinity with the stationary phase, thereby enabling their separation [52]. As they exit the column, the separated compounds enter the mass spectrometer, where they are ionized by electron impact. This ionization causes the molecules to fragment into characteristic ions. These ions are then separated based on their mass-to-charge ratio (*m*/*z*) using a charge analyser and subsequently detected to generate a mass spectrum specific to each compound [53]. The resulting spectrum consists of a molecular fingerprint, enabling the identification of volatile compounds through comparison with spectral databases. The combination of retention time data from GC and mass spectra (MS) gives GC-MS high analytical reliability [54].

GC-MS is widely recognized as a reference laboratory technique for the identification and quantification of VOCs generated during lipid oxidation in food systems [55]. Its high sensitivity and selectivity enable detailed characterization of oxidation products such as aldehydes, ketones, and hydrocarbons, which are important contributors to the volatile profiles associated with rancidity and quality deterioration. These oxidation-derived VOCs represent the chemical information detected as overall response patterns by e-nose systems. Therefore, GC–MS analysis is essential for identifying specific compounds responsible for changes in volatile fingerprints and for establishing relationships between VOC profiles and e-nose responses.

However, despite its analytical performance, GC-MS presents several limitations, including time-consuming sample preparation [56], the need for skilled operators [57], high operational costs, and lack of portability, making it unsuitable for rapid, on-site, or real-time monitoring in industrial environments [58]. These limitations have driven increasing interest in alternative analytical approaches, such as e-nose systems, which offer rapid, non-destructive, and near-real-time analysis of VOC profiles [59]. Although less specific than GC–MS, e-nose technologies provide a practical solution for continuous quality monitoring and early detection of lipid oxidation in complex food matrices. Therefore, GC-MS and e-nose systems should be considered complementary approaches, where GC-MS provides detailed chemical characterization of individual volatile compounds, while e-noses enable rapid assessment of global VOC patterns.

Beyond their individual applications, GC-MS and headspace solid-phase microextraction (HS-SPME) are increasingly integrated with e-nose systems as complementary analytical approaches for improving VOC-based prediction performance. While e-nose systems provide rapid responses to overall VOC patterns, HS-SPME-GC-MS enables the extraction, identification, and quantification of specific volatile compounds associated with quality changes, including lipid oxidation markers. This combination facilitates the correlation between sensor response patterns and individual VOC profiles, improving the interpretation of e-nose signals and enhancing the accuracy and reliability of predictive models. Therefore, the integration of e-nose technology with conventional analytical techniques represents a promising strategy for developing more robust and comprehensive food-monitoring systems [7].

### 3.2. High-Performance Liquid Chromatography (HPLC)

HPLC is widely used for the analysis of **non-volatile and semi-polar products** generated during lipid oxidation, particularly secondary oxidation products that cannot be detected by gas chromatography [60]. In lipid oxidation studies, HPLC is primarily applied to quantify carbonyl compounds and hydroperoxide-derived products, including malondialdehyde (MDA), 4-hydroxynonenal (4-HNE), 4-hydroxyhexenal (4-HHE) [61], and related reactive aldehydes, which are key biomarkers of oxidative degradation in food systems [62]. Because many of these compounds are highly reactive, unstable, and poorly detectable in their native form, HPLC analysis often requires a derivatization step prior to chromatographic separation [63]. For instance, carbonyl compounds such as MDA are commonly derivatized [64] with reagents like 2,4-dinitrophenylhydrazine (DNPH) to form stable hydrazone derivatives [65]. This step improves both detectability and chromatographic stability, enabling accurate quantification of lipid oxidation products in complex food matrices. Once derivatized, these compounds are separated and quantified by HPLC coupled with UV, fluorescence, or mass spectrometric detection, allowing for precise measurement of oxidative markers even at low concentrations [66]. This makes HPLC particularly suitable for monitoring the progression of secondary lipid oxidation, especially in edible oils, meat products, and dairy matrices [67].

However, despite its high sensitivity, selectivity, and robustness, HPLC remains a laboratory-based, offline analytical technique, requiring sample extraction, derivatization, and instrumental analysis [68]. Consequently, it is not suitable for real-time or in-line monitoring of lipid oxidation in industrial food-processing environments [69]. Nevertheless, HPLC remains an important reference method for validating rapid sensing technologies by providing quantitative information on specific oxidation markers and supporting the interpretation of sensor-based measurements.

### 3.3. Thiobarbituric Acid Reactive Substances (TBARS)

The formation of secondary products from lipid oxidation can be evaluated by determining the thiobarbituric acid reactive substances (TBARS). This method is based on the reaction between malondialdehyde (MDA), one of the main degradation products of lipid hydroperoxides, and thiobarbituric acid (TBA), leading to the formation of a pink-coloured complex whose intensity is proportional to the concentration of oxidized compounds present in the sample. Although MDA is the predominant compound quantified by this method, other carbonyl compounds derived from lipid oxidation can also react with TBA [70]. The TBARS test is thus a global indicator of secondary lipid oxidation and is commonly used to assess the oxidation state of edible oils and fats [71].

The results obtained from electronic-nose measurements should therefore be interpreted in relation to validated chemical indicators of lipid oxidation. Unlike TBARS or the peroxide value, which provide quantitative information regarding specific stages of oxidation, electronic noses generate a global fingerprint of volatile compounds released during oxidation. As lipid oxidation progresses, the formation of aldehydes, ketones, and other low-molecular-weight compounds modifies the overall sensor response pattern. Multivariate statistical approaches, such as partial least squares regression (PLSR), can then be applied to establish relationships between sensor signals and reference oxidation parameters. This approach allows for evaluation of whether changes detected by the electronic nose correspond to oxidative deterioration measured by conventional analytical techniques. Previous studies have shown that the electronic-nose responses can be significantly linked to secondary lipid oxidation products and TBARS values, supporting the use of sensor-based measurements in combination with conventional analytical methods for oxidation assessment [72]. Using PLS regression, significant relationships between the responses of MOS sensors and chemical indicators of lipid oxidation, including TBARS and volatile compounds such as hexanal, pentanal, and nonanal, can be determined, highlighting the potential of electronic noses to monitor oxidation-related changes [73].

## 4. Mini Electronic Nose (E-Nose)

### 4.1. Principle

E-nose systems are analytical devices designed to detect VOCs by global pattern recognition rather than molecular-level identification [74]. Figure 2 illustrates the analogy between the biological olfactory system and the e-nose technology used for VOC analysis. The biological olfactory system detects odours through a complex interaction between odorant molecules and olfactory receptor neurons located in the olfactory epithelium. When VOCs enter the nasal cavity, they dissolve in the mucus layer and bind to olfactory receptors located on the cilia of sensory neurons [75]. This interaction generates electrical signals that are transmitted to the olfactory bulb and then interpreted by the olfactory cortex, where the brain recognizes the overall activation pattern as a specific odour rather than identifying individual molecules separately [76]. This biological mechanism for global pattern recognition is the fundamental inspiration behind e-nose technology. An e-nose mimics this natural olfactory process by using networks of partially selective chemical sensors capable of interacting with several VOCs simultaneously [77]. Unlike chromatographic techniques, e-nose systems generate a collective response model corresponding to the overall composition of the volatile mixture [78]. Each sensor within the network reacts differently depending on its physico-chemical sensitivity, producing electrical variations that collectively form an olfactory digital signature characteristic of an olfactory fingerprint [79]. This signal pattern reflects the overall VOC profile of the analysed sample, then is processed using chemometric methods and artificial intelligence.

An **e-nose** usually consists of two main components: a hardware unit and a software unit. The hardware part generally includes a sampling system, a chamber containing gas sensor arrays, signal conditioning circuits, microcontrollers and wireless communication modules for real-time analysis. The software component is responsible for data acquisition, preprocessing, feature extraction, pattern recognition, and classification using chemometric algorithms or artificial intelligence [80]. The automated detection process begins when volatile compounds released from a sample interact with the sensor array, producing a specific electrical response pattern often referred to as a digital aromatic signature or scent fingerprint. These signatures are compared to a previously collected library (dataset) of reference aroma profiles stored in the system’s database [81]. During the training phase, the e-nose is exposed to known samples to build this dataset and train machine learning models capable of recognizing characteristic VOC patterns [82]. During analysis, the newly acquired signal is automatically processed and compared to stored signatures using statistical and machine learning algorithms such as principal component analysis (PCA), linear discriminant analysis (LDA), support vector machines (SVMs), or artificial neural networks (ANNs) [83]. The system can then classify, identify, or predict the sample’s quality status based on similarity to the reference database.

**Figure 2:**
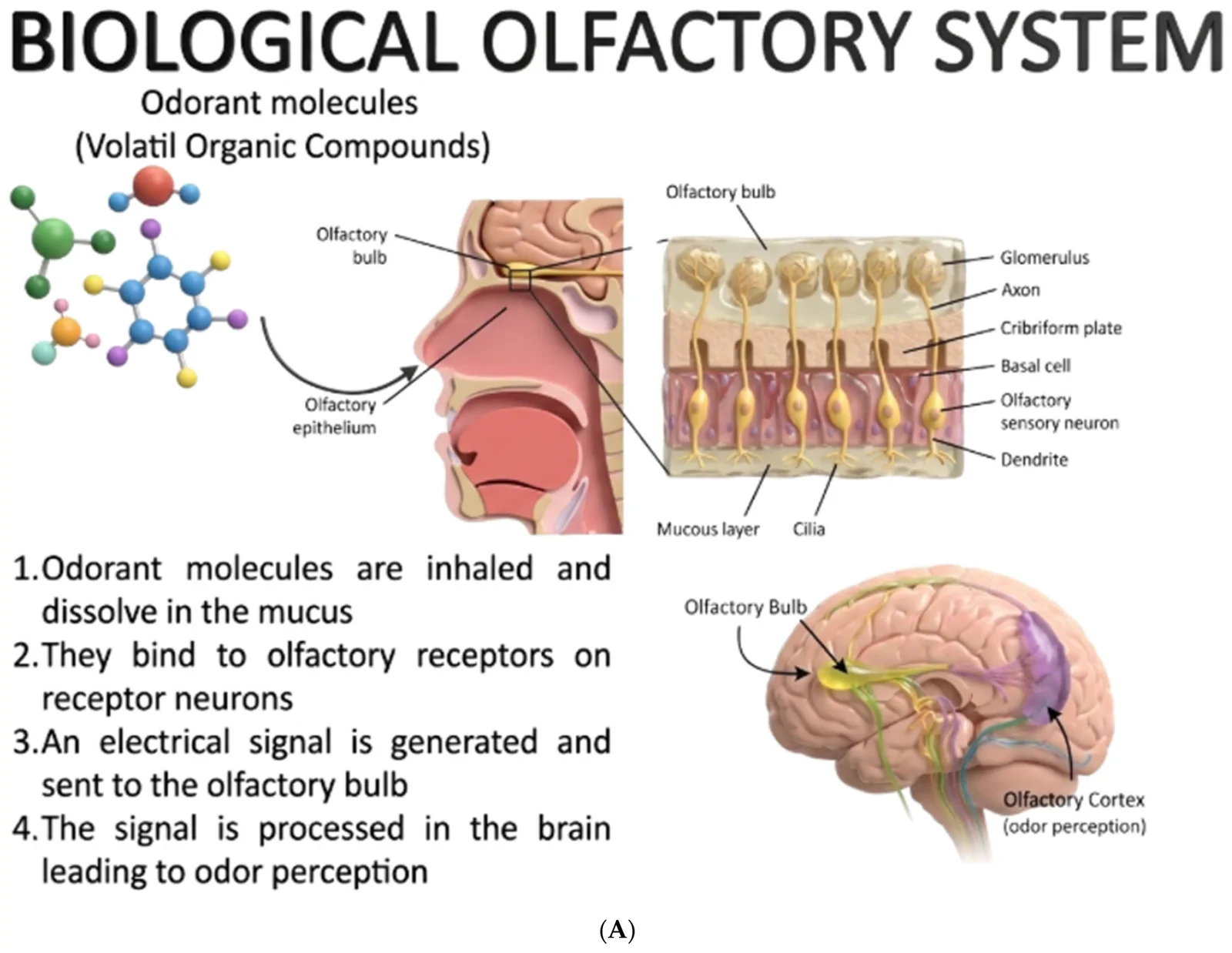

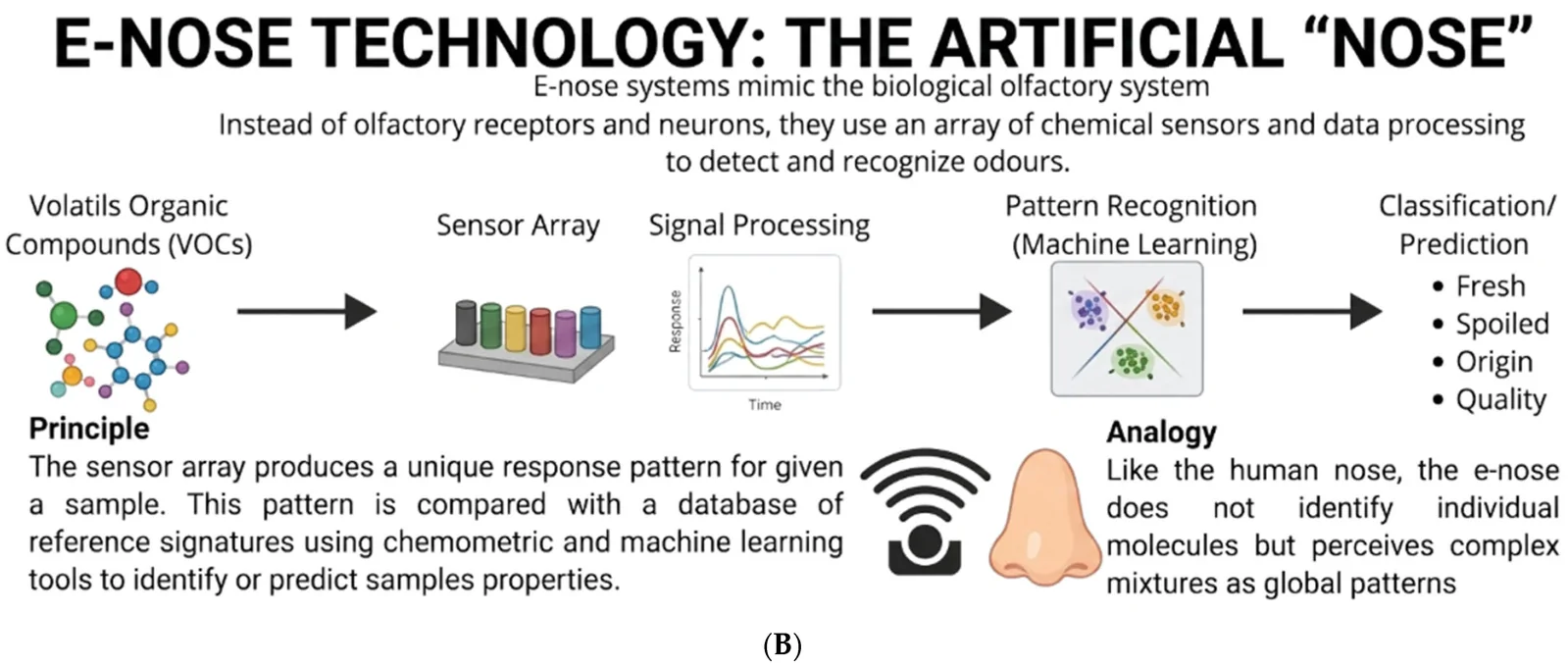
Analogy between human olfactory perception (**A**) and electronic nose systems (**B**). Redrawn by the authors based on Refs. [84,85]. Created in BioRender (https://www.biorender.com/).

### 4.2. Sensor Mechanism

The e-nose detection mechanism is based on networks of non-specific, partially selective chemical sensors [86], most often metal oxide semiconductor (MOS) sensors [87]. These sensors respond to a wide range of VOCs, resulting in an overlap of sensitivity schemes across the network [11]. When VOCs interact with the sensor surface, they induce physico-chemical changes such as variations in electrical resistance or conductivity due to redox reactions occurring [88]. Each sensor has a different response intensity depending on the chemical properties of the VOC mixture, and the combination of all responses forms a single signal pattern. Each sensor produces a non-specific but reproducible response to several compounds, which is essential for generating discriminatory fingerprints [89]. This cross-reactivity is a deliberate design feature, as it allows the system to capture the complexity of real-world odour mixtures, including those generated during lipid oxidation [90].

It is important to note that individual sensors are generally non-specific and can react simultaneously with many VOCs. However, the reproducibility of their collective response pattern allows for the generation of discriminant odour fingerprints capable of differentiating complex volatile mixtures [91]. This cross-reactivity is a deliberate design feature that allows e-nose systems to capture the complexity of real-world odours, including food flavours, fermentation products, environmental pollutants, and subproducts of lipid oxidation [92].

Environmental parameters such as temperature, relative humidity, airflow and sensor drift can significantly influence sensor performance and signal stability [93]. Humidity is particularly critical because water molecules can compete with VOCs for adsorption sites on the sensor surface, thus modifying the sensor’s sensitivity and base resistance. To improve analytical reliability [94], modern e-nose systems frequently incorporate moisture compensation models, sensor calibration procedures, basic correction algorithms and drift compensation techniques based on machine learning approaches.

### 4.3. System Architecture

An e-nose system integrates several functional components that work sequentially to transform chemical information into interpretable data. As illustrated in Figure 3, an electronic nose typically comprises a sampling system for controlling VOC delivery, a sensor array, a data acquisition unit, and a pattern recognition module. The sensor array generates electrical signals in response to VOC exposure, which are then digitalized and processed [95]. Modern e-nose architectures are increasingly integrating embedded electronics and artificial intelligence modules, allowing for real-time data acquisition and on-device processing [96].

Today, e-nose systems have integrated platforms combining sensor networks with computational algorithms to produce and interpret odour fingerprints, highlighting their evolution [97].

### 4.4. Types of Sensors Used in E-Noses

E-noses rely on a variety of sensor technologies, each based on distinct physico-chemical transduction mechanisms for VOC detection, as can be seen in Figure 4. These sensors differ in their sensitivity, selectivity, response time and operating principles, which directly influence the nature of the signals generated [57]. Table 2 presents the main sensor technologies used in e-noses as well as their analytical performance and food applications. The detection ranges show the sensitivity of each type of sensor to VOCs [98].

MOSs and electrochemical sensors are among the most widely adopted sensing technologies due to their robustness and cost-effectiveness, whereas conducting polymer-based sensors are particularly valued for their rapid analysis of aromatic compounds. quartz crystal microbalance (QCM) and surface acoustic wave (SAW) sensors provide high sensitivity through the detection of mass or frequency variations, while field-effect transistor (FET)-based and optical sensors enable the detection of extremely low analyte concentrations, especially in advanced application domains. Understanding these types of sensors is essential, as they determine not only how VOCs are detected, but also the complexity and multidimensional nature of the data obtained [99], which ultimately requires advanced chemometric and machine learning approaches for interpretation [100].

**Table 2:** Comparison of sensor technologies used in miniaturized electronic-nose systems for food-quality monitoring.

Sensor Technology	Sensing Principle	Detection Range/LOD	Selectivity	Response Time	Operating Conditions	Stability	Advantages	Limitations	Recommended Applications	References
Metal oxide semiconductor (MOS)	Change in electrical resistance due to VOC adsorption and surface reactions	5–30 ppb	>100 often requires pattern recognition algorithms	Seconds to minutes	Usually requires elevated operating temperature (100–400 °C)	Response may change over weeks to months; requires periodic recalibration	High sensitivity, low cost, robust, easy miniaturization, widely used in mini e-noses	High power consumption due to heating; cross-sensitivity; baseline drift	Real-time oxidation monitoring, oils, meat, dairy, industrial quality control	[101]
Conducting polymer (CP)	Change in electrical conductivity after VOC absorption into polymer matrix	<10 ppb	2 to 10 depending on polymer chemistry; moderate sensitivity	Seconds to minutes	Operates mainly at room temperature (20–30 °C)	Limited long-term stability due to aging and humidity effects	Low power consumption, room-temperature operation, easy fabrication	Poor mechanical stability; sensitivity to environmental conditions	Portable food-monitoring devices	[91]
Quartz crystal microbalance (QCM)	Frequency shift caused by VOC adsorption on coated quartz crystal	ppb–ppm,	10 to 100 depending on coating materials	Seconds to a few minutes	Room temperature	Good stability but affected by coating degradation	High sensitivity, low operating temperature	Requires specialized coatings; humidity interference	VOC fingerprint analysis and research applications	[102]
Electrochemical sensors	Electrochemical reaction between VOCs and sensing electrode	Sub-ppm to ppm, depending on the target analyte	Good selectivity depending on electrode materials	Seconds to <1 min	15–40 °C	Moderate to good stability	Low power consumption, compact size, good sensitivity	Limited VOC range; electrode aging	Targeted VOC detection and portable monitoring	[103]
Optical sensors	Changes in optical properties after VOC interaction	ppb–ppm	10 to 100 depending on sensing material	Milliseconds to minutes	Room temperature	Good stability	Low power consumption, immune to electromagnetic interference	Expensive optical components; complex design	Advanced portable sensing systems	[104]
Field-effect transistor (FET) sensors	VOC-induced changes in semiconductor channel conductivity	ppb–ppm	5 to 100 depending on the semiconductor and surface functionalization.	Milliseconds to seconds/minutes	Room temperature to <100 °C,	Requires further validation for long-term use	Extremely small size, low power, integration with electronics	Fabrication complexity; environmental sensitivity	Future wearable and IoT-based monitoring	[105]
**Surface acoustic wave (SAW) sensors**	Changes in acoustic wave propagation caused by VOC adsorption	ppb–ppm	2 to 20, with values potentially exceeding 100	Milliseconds to seconds	Room temperature	Good but affected by humidity and coatings	High sensitivity, rapid detection, miniaturization potential	Expensive fabrication; coating stability issues	High-performance portable VOC monitoring	[106]

Compared to conventional analytical methods commonly used for the evaluation of lipid oxidation, mini e-noses offer several advantages, notably in terms of speed, portability and the ability to provide real-time monitoring of volatile organic compound (VOC) models. Traditional techniques, such as GC–MS, HPLC, peroxide index (PV), TBARS, and Rancimat analysis, provide accurate and specific measurements of oxidation markers and remain essential reference methods for mechanistic studies and validation. However, these approaches often require complex sample preparation, laboratory equipment, trained operators and destructive measurements, which limits their application for continuous and on-site monitoring [107]. On the other hand, mini e-noses allow for rapid, non-destructive and cost-effective analysis by detecting global changes in VOC profiles associated with lipid degradation. However, unlike GC–MS, mini e-noses generally lack molecular specificity and require advanced chemometric models to interpret the complex sensor responses and correlate them with oxidation parameters. Therefore, mini e-noses should be considered complementary tools rather than substitutes for traditional analytical methods. As summarized in Table 2, the integration of a mini e-nose with reference techniques can provide both rapid screening capabilities and reliable quantitative information on the progression of lipid oxidation, thus supporting their application in intelligent packaging, food-quality control, and real-time monitoring systems.

#### 4.4.1. Metal Oxide Semiconductor (MOS) Sensors

MOSs are among the most widely used detection technologies in e-nose systems [108]. They operate based on changes in electrical resistance when the VOCs interact with the surface of the sensing material [109]. Generally fabricated of metal oxide semiconductors, such as tin oxide (SnO_2_) or zinc oxide (ZnO) [110], these sensors function through processes and surface reaction involving gaseous molecules and oxygenated species adsorbed onto the sensor surface. This interaction leads to a modulation of the charge carrier density, leading to measurable variations in resistance [111]. MOSs are particularly attractive due to their high sensitivity and specificity, robustness, fast response, small size, low cost, and ease of use and integration into sensor networks [112].

In addition, they can operate at high temperatures and are suitable for a wide range of gases. As a result, the signals generated are highly transverse and multidimensional, making direct interpretation difficult. Therefore, advanced chemometric techniques are required to extract relevant features and distinguish complex mixtures [113]. However, these approaches also have several limitations, including reduced accuracy, sensitivity to humidity, high energy consumption, and susceptibility to sulphur poisoning, which limit their overall performance [98].

#### 4.4.2. Electrochemical Sensors

Electrochemical sensors operate according to the redox reactions that occur on the surface of an electrode when it is exposed to target gases [114], which makes it possible to detect changes in electrical properties, such as current or voltage, resulting from chemical reactions between the target analyte and the electrode surface [115]. The latter are proportional to the VOC concentration [116]. Due to their high sensitivity, selectivity, robustness and low energy requirements, electrochemical sensors are extensively used for the detection of specific compounds [117].

There are different types of electrochemical sensors, such as amperometry, potentiometric and conductometric sensors. Amperometry sensors measure the current produced by the oxidation–reduction reaction on the surface of the electrode, while potentiometric sensors measure the voltage generated by this same reaction [118]. Conductometric sensors detect changes in electrical conductivity caused by interactions between the target analyte and the sensing material.

Depending on the sensor design and target compounds, detection limits may range from the low parts-per-billion (ppb) level to several parts per million (ppm), making them suitable for monitoring trace VOCs associated with food spoilage, oxidation, fermentation, and contamination [119]. Compared with MOS sensors, electrochemical sensors generally exhibit superior selectivity toward specific gases such as ethanol, ammonia, hydrogen sulphide, sulphur-containing compounds, nitrogen oxides, and carbon monoxide. They also provide fast response and recovery times, often within a few seconds to minutes, which is advantageous for real-time monitoring applications.

Consequently, electrochemical sensors have been incorporated into mini e-nose systems for assessing fruit ripeness, monitoring meat and fish freshness, detecting milk spoilage, and evaluating oxidation processes in edible oils [120].

#### 4.4.3. Conducting Polymer Sensors

Conductometric sensors, particularly those based on conducting polymers such as polyaniline, detect VOCs through changes in electrical conductivity [121] and modify their electrical properties according to variations in their environment, such as exposure to certain gases or chemicals [122]. When gas molecules interact with the sensor surface, they induce charge transfer processes that change the electrical resistance of the material. These sensors are widely used because of their simplicity, low cost, sensitivity to many gases, and fast response time [123].

In addition, they are resistant to sensor poisoning and can be used at room temperature, which eliminates the need for a heated part. Their detection performance is strongly influenced by their nanostructure, as an increased surface/volume ratio improves gas adsorption and sensitivity [124]. Environmental factors such as humidity and temperature can affect their stability and reproducibility. However, they also have a limited and shorter lifespan than other sensors and can be affected by drift [125].

#### 4.4.4. Quartz Crystal Microbalance Sensors

QCM sensors are used in e-nose systems due to their high sensitivity to mass and mechanical changes on the sensor surface. These sensors operate based on the piezoelectric effect, where mechanical changes induced by the adsorption of VOCs are transduced into measurable electrical signals [126]. Moreover, piezoelectric sensors are known for their simplicity, fast response, low cost, ease of miniaturization and low detection limit, allowing a broad spectrum to be obtained [127].

Moreover, the sensitivity and selectivity of these networks can be modulated through the careful selection of chemically sensitive, adsorbed layers [128]. Gas sensors based on QCM technology operate by detecting changes in mass through monitoring variations in resonance frequency [129]. The measurement of this frequency variation makes it possible to quantify the mass or concentration of the adsorbed gas [130]. This device can also function as a gas and humidity sensor [131], owing to its high sensitivity and stability, and, in particular, its low operating temperature range (0–50 °C).

The resistance of the multi-sensor module can be mechanically described using a mass–spring–damper model. But these sensors are difficult to implement, can be sensitive to humidity and temperature, and have a low signal-to-noise ratio, which indicates that the useful signal is drowned in the background noise, making the data unclear [132].

#### 4.4.5. Optical Sensors

Optical sensors detect VOCs by monitoring changes in optical properties, such as light absorption, fluorescence or scattering, when gas molecules interact with the sensing material [133]. Fluorescence spectroscopy is also widely used for VOC detection [134]. It is based on the excitation of fluorophores and their emission of photons, with the fluorescence intensity being affected by elements such as temperature, solvent and pH [135].

Organic fluorophores, including porphyrins and other functional dyes, are perfectly suited for the detection of various VOCs due to their biocompatibility and flexibility and their ability to interact selectively with different VOCs [136]. These sensors offer high sensitivity that generates detectable signals upon contact with the target analyte [137] and fast response times, as well as low cost and light weight, which is particularly useful in applications requiring remote sensing or no contact [138]. They are also immune to electromagnetic interference waves, which makes them a great advantage. On the other hand, they are difficult to implement, have low portability, and may be affected by light interference [139].

#### 4.4.6. Field-Effect Transistor (FET) Sensors

FET sensors operate by detecting changes in the electrical properties of a semiconductor channel when exposed to gas molecules [140]. The interaction between VOCs and the sensing layer modulates the charge carrier density, thereby altering the current flowing through the device [141]. FETs operate through a mechanism involving the adsorption of gas on the sensitive material, which induces variations in the gate potential or charge distribution [142]. This modulation affects the carrier density in the semiconductor channel, leading to measurable current or voltage variations. The latter are designed to control the variations in the output signals and to calibrate the variations in environmental conditions during detection, such as humidity and temperature.

Thus, FET sensors can be effectively used for the implementation of practical and high-precision gas detection systems [143]. FET-based sensors are known for their high sensitivity and compatibility with nanoscale materials, making them promising for advanced e-nose applications [144]. Nevertheless, their responses are often nonlinear and influenced by multiple factors, requiring sophisticated modelling approaches for accurate interpretation.

#### 4.4.7. Surface Acoustic Wave (SAW) Sensors

SAW is an important class based on the propagation of mechanical waves through a piezoelectric material to detect VOCs. Among them, surface acoustic wave sensors are the most widely used [145]. These sensors operate based on the piezoelectric effect, where the interaction between VOC molecules and a sensitive coating deposited on the sensor surface induces changes in mass, elasticity or conductivity, resulting in measurable variations in the wave speed or resonance frequency [146]. This mechanism allows for very sensitive detection, often at very low concentration levels, with fast response times and real-time monitoring capabilities [147].

SAW sensors are known for their high sensitivity, small size and low cost due to the confinement of acoustic energy to the surface, making them particularly suitable for gas detection applications [148]. Moreover, they can respond to all gases [149]. However, SAW sensors generally have limited selectivity, as their response depends on the physico-chemical properties of the adsorbed molecules and the sensitive layer. Moreover, their performance can be affected by environmental factors such as temperature, leading to signal drift and variability. They are also difficult to implement and have a low signal-to-noise ratio [150].

### 4.5. Commercially Available E-Nose

A growing number of mini e-nose systems have become commercially available in recent years, reflecting advances in sensor miniaturization, embedded electronics, and data-processing capabilities [78]. These systems differ considerably in terms of sensor technology, analytical performance, portability, data-processing capability, and suitability for industrial food applications. Table 3 summarizes representative commercial e-nose platforms and their applications in food-quality monitoring.

The PEN series developed by AIRSENSE Analytics GmbH is among the most widely used mini e-nose systems in food research. The PEN 2 and PEN 3 models are based mainly on metal oxide semiconductor (MOS) sensor arrays and integrate pattern recognition algorithms for VOC fingerprint analysis. The PEN 3 contains 10 different sensors and is characterized by its compact size, portability, and robustness, making it suitable for applications such as food-quality control, spoilage detection, and aroma profiling in products including wine, coffee, and meat [151]. However, like other MOS-based systems, its performance can be affected by humidity variations, sensor drift, and the need for regular calibration, which may limit long-term industrial deployment.

Alpha MOS provides several commercial sensory analysis platforms, including the FOX series and HERACLES Neo [152]. These systems combine advanced sensor arrays with dedicated data-analysis software for aroma characterization, classification, and quality evaluation. They have been applied in sensory analysis, wine classification, dairy- and meat-quality assessment, beverage analysis, and product authentication. The HERACLES Neo, which uses ultra-fast gas chromatography technology, provides improved compound separation compared with conventional sensor-array systems; however, the increased analytical capability is associated with higher instrument complexity and cost, which may restrict its use for continuous in-line monitoring.

Aryballe has developed biomimetic olfactory systems, including the NeOse Pro and Sense Kit, which combine chemical sensing with machine learning-based aroma recognition. These systems have demonstrated potential for aroma fingerprinting, product authentication, and food and beverage-quality assessment [153] Their compact design and digital approach to aroma analysis provide advantages for applications. Nevertheless, further validation under variable environmental conditions and across diverse food matrices is required before widespread industrial implementation [154].

Sensigent has developed several e-nose platforms, including the eNose MSEM 1400, eNose Aqua OEM, eNose QA, and Cyranose 320. These systems have been investigated for applications including contamination detection, off-flavour monitoring, and quality assurance in beverages and other products. Their portability and flexible sensor configurations provide advantages for field applications; however, sensor stability and calibration requirements remain important considerations for long-term operation.

Other commercially available systems have also demonstrated potential for food-quality monitoring. The zNose series developed by Electronic Sensor Technology applies fast gas chromatography-based detection for flavour compound analysis and contamination monitoring. Although these systems provide high analytical specificity, their complexity and cost may limit widespread industrial implementation. Similarly, Tellspec has developed compact spectroscopic sensing platforms aimed at rapid food analysis, including freshness and contamination assessment, although further validation is required for routine industrial applications.

Recent developments have also focused on highly integrated and connected e-nose platforms. Systems such as the e-nose MX from Stratuscent and semiconductor-based sensors developed by Bosch Sensortec demonstrate the potential of low-cost VOC monitoring and integration with smart devices and Internet of Things (IoT) platforms. These systems offer advantages in terms of miniaturization, low power consumption, and connectivity; however, challenges related to selectivity, calibration transfer, and long-term stability remain important barriers for industrial food monitoring.

Despite the increasing commercial availability of mini e-nose systems, their analytical performance and industrial applicability vary considerably depending on the sensor technology, data-processing capability, and intended application. MOS-based devices, such as the PEN series, are widely used because of their robustness, relatively low cost, and compatibility with portable platforms. However, they remain susceptible to sensor drift, humidity interference, and cross-sensitivity, requiring regular calibration for reliable long-term operation. Optical and hybrid sensing systems generally provide improved selectivity and stability but are often associated with higher costs and more complex instrumentation. Consequently, the selection of a commercial e-nose should consider not only analytical performance but also practical factors such as portability, maintenance requirements, operating conditions, and the specific food matrix being analysed.

Overall, commercially available e-nose systems provide valuable tools for rapid VOC fingerprint analysis and quality assessment. However, their suitability for industrial food applications depends not only on analytical performance but also on factors such as sensor stability, calibration requirements, maintenance, cost, and robustness under real processing conditions. Therefore, continued improvements in sensor materials, data-processing algorithms, and validation protocols are required to facilitate the transition of commercial e-nose technologies from laboratory applications to reliable industrial monitoring platforms.

### 4.6. Applications of E-Noses in Food-Quality Control

E-noses have been applied to a wide variety of foods, especially those rich in fat [7]. Vegetable oils, such as olive oil, are widely studied for quality monitoring, oxidation assessment, and aging analysis. The objective is to characterize edible oils and monitor their compositional and sensory evolution during storage [90]. Meat products, dairy products, fermented products and beverages also represent important application areas for the mini e-nose [85]. In these matrices, lipids play a major role in aroma formation, since lipid oxidation and degradation generate volatile organic compounds (VOCs) such as aldehydes, ketones, alcohols, and hydrocarbons, which can be detected by e-nose [154].

Mini e-noses could play an important role in the development of smart food packaging and intelligent food supply chains by providing continuous information on VOC patterns associated with freshness, spoilage, and lipid oxidation. Embedded or externally coupled e-nose sensors could monitor changes in the package headspace and transmit measurements through IoT, or wireless communication systems, enabling real-time quality tracking from storage and transportation to retail. Such systems could complement conventional indicators by providing non-destructive information directly related to the volatile state of the product [155]. In an Industry 4.0 framework, e-nose data could be combined with temperature, humidity, oxygen, and traceability data and processed using machine learning algorithms to support automated quality assessment, early spoilage detection, and predictive decision-making. These data streams could also serve as inputs to digital twins of food products, packages, or supply-chain processes, allowing the virtual representation to be continuously updated with real-time sensor information and used to predict quality evolution under different storage and transportation conditions. Recent research indicates that digital twins are increasingly being considered for real-time monitoring, simulation, and quality prediction within food processing and supply chains [156]. However, practical implementation still requires improvements in sensor stability, wireless connectivity, data standardization, model transferability, and validation under realistic supply-chain conditions.

The development of miniaturized, low-power sensor systems also creates opportunities for integration within the Industry 4.0 framework. Unlike conventional laboratory instruments, mini e-noses can potentially be connected to wireless communication systems and Internet of Things (IoT) networks, allowing sensor data to be transmitted continuously or periodically during food storage, processing, and distribution. When combined with environmental variables such as temperature, humidity, oxygen concentration, and storage time, VOC sensor data could contribute to data-driven quality-control systems. Machine learning models could then be used to detect abnormal changes, classify product quality, or predict the progression of oxidative deterioration. Another promising future direction involves integrating mini e-noses into digital-twin frameworks to enable real-time monitoring and prediction of food quality and oxidative deterioration [157]. In principle, a digital twin could combine real-time or repeated sensor measurements with mathematical or data-driven models representing changes occurring in a food product during processing or storage. For lipid-containing foods, VOC fingerprints obtained from a mini e-nose could potentially be integrated with information on temperature history, oxygen exposure, humidity, and conventional oxidation markers to estimate the current oxidative state or predict the remaining shelf life. However, this concept remains largely prospective for food-related mini electronic noses. Its practical implementation will require robust sensor calibration, drift compensation, standardized data acquisition, reliable data transmission, and validated predictive models capable of operating across different products and environmental conditions [158].

Overall, the value of mini e-noses within future Industry 4.0 and digital-twin systems lies primarily in their ability to provide frequent, low-power, and potentially connected measurements of VOC patterns. Rather than replacing high-specificity analytical techniques, mini e-noses could act as distributed sensing devices that complement laboratory reference methods. Future research should therefore focus on hybrid monitoring strategies combining conventional analytical characterization, miniaturized VOC sensors, IoT connectivity, and predictive modelling to support more continuous and data-driven food-quality management [159].

#### 4.6.1. Meat

Meat and seafood products are particularly susceptible to lipid oxidation because of their content of unsaturated fatty acids, phospholipids, and pro-oxidative components such as haem iron. During storage and thermal processing, lipid hydroperoxides decompose into secondary oxidation products, including aldehydes, ketones, alcohols, hydrocarbons, and furans. Among these compounds, hexanal is one of the most frequently investigated volatile markers because it is commonly associated with the oxidation of linoleic acid and contributes to rancid and off-flavour development in muscle foods. Other compounds, including pentanal, heptanal, octanal, nonanal, and 2-pentylfuran, may also contribute to the volatile fingerprint associated with oxidative deterioration. Recent work on chicken meat further confirms that aldehydes constitute an important group of volatile compounds associated with lipid-derived flavour and off-flavour formation, with hexanal being among the most abundant oxidation-related compounds [160].

A relevant example of an artificial olfactory system applied directly to lipid oxidation was reported by Kim et al. (2016) [161], who developed a colorimetric sensor array for predicting warmed-over flavour development in cooked chicken. The system was evaluated against conventional oxidation indicators, including TBARS and the oxidation-related aldehydes pentanal, hexanal, and heptanal. The sensor responses were strongly associated with these markers, and partial least squares regression models successfully predicted their concentrations. This study is particularly relevant because it demonstrates a direct relationship between sensor-array signals and chemically validated secondary lipid oxidation products rather than relying solely on the classification of storage time or general freshness [161].

More recently, Wang et al. investigated the evolution of beef flavour during postmortem aging using sensory evaluation, electronic-nose analysis, and GC-MS. The study identified several important volatile compounds, including hexanal, heptanal, octanal, nonanal, decanal, pentanal, dodecanal, and 2-pentylfuran. The authors identified lipid oxidation as an important pathway contributing to volatile formation during beef aging. These results illustrate the potential of combining e-nose measurements with compound-specific analysis to improve the interpretation of sensor responses and to distinguish volatile changes associated with lipid oxidation from other biochemical processes occurring during storage [162].

Recent developments have also demonstrated the potential value of combining e-nose analysis with lipidomics. They investigated chicken breast meat with different levels of intramuscular fat using targeted lipidomics and e-nose technology during cold storage. Differences in the aroma profile were associated with aldehydes, ketones, alkanes, and other volatile compounds, while lipidomic analysis revealed relationships between specific lipid classes and aroma characteristics. Although the main objective of this study was not exclusively the monitoring of lipid oxidation, it demonstrates how sensor data can be interpreted in relation to the underlying lipid composition and its evolution during storage. This combined approach could be particularly valuable for future mini e-nose applications designed to monitor oxidation-related changes in complex meat matrices [163].

Overall, recent studies demonstrate that artificial olfactory systems can provide useful information about oxidative changes in meat when their responses are associated with validated chemical markers. However, microbial spoilage and lipid oxidation may occur simultaneously and generate overlapping volatile fingerprints. Therefore, future mini e-nose applications should combine sensor responses with reference measurements such as TBARS, peroxide value, fatty acid composition, and GC-MS analysis of key oxidation-derived aldehydes. Such approaches would improve the specificity of sensor-based systems for real-time monitoring of oxidative deterioration in meat and seafood products.

#### 4.6.2. Dairy Products

Dairy products containing substantial amounts of milk fat, particularly cheese and butter, may undergo oxidative deterioration during storage and exposure to oxygen or light. Lipid oxidation can result in the degradation of unsaturated fatty acids and the formation of primary and secondary oxidation products, ultimately contributing to changes in flavour, aroma, and shelf life. However, the application of recent miniaturized e-nose technologies specifically validated for lipid oxidation monitoring in dairy products remains limited. This represents an important research gap because many existing electronic-nose studies on dairy products focus instead on authentication, product classification, fermentation, or general quality assessment.

Recent work nevertheless confirms that lipid oxidation is an important cause of quality deterioration in dairy matrices. The study of lipid and protein oxidation was carried out in vacuum-packed or modified atmosphere grated Kefalotyri cheese stored under retail presentation conditions. During storage, significant decreases in monounsaturated and polyunsaturated fatty acids were observed, while lipid hydroperoxides and malondialdehyde measurements confirmed oxidative deterioration. The results also demonstrated that storage conditions influenced both oxidation and sensory characteristics. Although this study did employ an e-nose, it identifies a suitable framework for the development of VOC-based monitoring approaches in dairy products [164]. Further recent work on Kefalotyri cheese has also demonstrated that storage conditions, particularly exposure to fluorescent light, can influence the oxidative status of dairy products. These findings reinforce the importance of monitoring oxidation in high-fat dairy matrices during refrigerated storage and retail display. The formation of oxidation products is expected to modify the headspace composition, suggesting that compact sensor arrays could potentially provide rapid and non-destructive information on quality deterioration [165].

However, the interpretation of electronic-nose responses in dairy products requires caution. Aldehydes and ketones detected in the headspace may originate from lipid oxidation, but they can also be generated through lipolysis, microbial metabolism, fermentation, enzymatic activity, and cheese ripening. Consequently, an e-nose model that simply distinguishes samples according to storage time or product type cannot automatically be considered a lipid oxidation model.

Future studies should therefore specifically investigate whether mini e-nose responses can be correlated with validated markers such as lipid hydroperoxides, TBARS or MDA, peroxide value, and oxidation-derived volatile compounds determined by GC-MS or GC-IMS. Such experiments would help identify which sensor patterns are specifically associated with oxidative deterioration. Consequently, fat-rich dairy products represent a promising but still underexplored application area for mini electronic noses, and the limited number of recent studies directly combining sensor arrays with validated lipid oxidation markers highlights an important opportunity for future research.

#### 4.6.3. Fermented Products

Fermented and processed foods represent particularly complex matrices for the application of electronic noses because their volatile profiles can be influenced simultaneously by lipid oxidation, microbial metabolism, enzymatic reactions, thermal processing, and Maillard chemistry. Consequently, changes in an electronic-nose fingerprint cannot automatically be attributed to oxidative deterioration. For this reason, only studies involving validated oxidation markers or clearly identified oxidation-derived VOCs should be considered when evaluating the potential of mini e-nose systems for lipid oxidation monitoring.

Wine provides an example of the application of recent low-cost electronic-nose technology to quality defects. Researchers developed a low-cost e-nose combined with near-infrared spectroscopy and machine learning modelling to classify wine faults. The study demonstrated that compact sensor technologies can successfully discriminate complex volatile patterns associated with different quality defects. However, because the study focused on wine-fault classification rather than specifically on lipid oxidation, it should not be considered direct evidence of oxidation monitoring. Instead, it illustrates the ability of low-cost electronic noses to analyse complex processed-food headspaces and highlights the importance of developing models specifically targeted at chemically validated oxidation markers [166].

Fermented meat products represent another relevant application of electronic-nose technology because lipid oxidation occurs simultaneously with fermentation and contributes to the development of volatile compounds. A recent review of e-nose applications in fermented foods reported the use of a PEN3 system equipped with ten MOS sensors to evaluate lipid oxidation in Chinese-style sausage during processing and storage. The volatile profile of fermented meat products may include oxidation-related compounds such as hexanal, together with alcohols, esters, sulfur compounds, and other fermentation-derived VOCs. These results highlight the potential of sensor arrays to monitor oxidative changes in fermented meat products, although complementary chemical analyses remain necessary to distinguish lipid oxidation products from compounds generated by microbial metabolism [167].

Recent research has further demonstrated the close relationship between oxidation and volatile evolution in dry-fermented sausages. In a 2026 study, the effect of millet-immobilized probiotic cultures on oxidation and volatile profiles was monitored during ripening and extended storage. The millet-containing formulations exhibited an antioxidant effect and reduced lipid oxidation, while the volatile profile became increasingly dominated by aldehydes and alcohols during storage. This study highlights the importance of monitoring oxidation-related volatile changes in fermented meat products and provides relevant target VOC families for future mini electronic-nose applications [168].

Beer provides another relevant example of oxidative deterioration in fermented foods. It has been demonstrated that beer aging is associated with the progressive accumulation of staling aldehydes, including furfural and trans-2-nonenal. The use of novel ***Saccharomyces cerevisiae*** variants reduced the accumulation of these compounds and improved flavour stability for up to 12 months. Although electronic-nose measurements were not used in this study, the results identify relevant volatile targets for the development of sensor-based systems designed to monitor oxidative aging and shelf life in beer [169].

More generally, the main challenge in fermented and processed foods is distinguishing VOCs produced by different chemical and biological pathways. Aldehydes and ketones can potentially originate from lipid oxidation but may also be generated during fermentation, microbial metabolism, thermal treatment, or amino acid degradation. Therefore, the use of general aroma classification models without chemical validation may lead to incorrect attribution of sensor responses. Thus, fermented and processed foods should be considered promising but analytically challenging matrices. Their complex VOC composition requires robust chemometric analysis and chemical validation before an electronic-nose response can be interpreted as an indicator of lipid oxidation. Future miniaturized systems may benefit from combining broadly responsive sensors with more selective sensing elements and complementary analytical methods to improve discrimination between oxidation-related VOCs and compounds generated by fermentation or processing.

#### 4.6.4. Vegetable Oils

Edible oils represent one of the most suitable matrices for monitoring lipid oxidation using miniaturized electronic noses because the major changes occurring during storage and heating are directly related to lipid degradation. The oxidation of unsaturated fatty acids generates hydroperoxides, which subsequently decompose into volatile compounds such as aldehydes, ketones, alcohols, hydrocarbons, and other oxidation products. These changes modify the headspace composition and contribute to rancidity, off-flavours, and progressive quality loss.

A recent study investigated the quality deterioration in soybean and canola oils subjected to repeated frying using an e-nose and e-tongue together with conventional chemical analyses. The authors measured oxygen induction time, acid value, *p*-anisidine value, malondialdehyde, and total polar compounds. These chemical indicators showed progressive deterioration with repeated frying. E-nose analysis also revealed changes in the volatile profile, including compounds such as 2-heptenal and 2,4-pentanedione. The study demonstrated that the evolution of sensor-detected volatiles could be interpreted together with conventional lipid oxidation parameters to evaluate the degradation of frying oils [170].

Olive oil is another particularly relevant application because rancidity is directly associated with the formation of oxidation-related volatile compounds. Gonzalez Viejo and Fuentes developed a low-cost e-nose system to assess different levels of rancidity in commercial extra-virgin olive oils. The samples were analysed using GC-MS, near-infrared spectroscopy, and a low-cost e-nose, and machine learning models were developed to classify rancidity levels and predict the abundance of aroma compounds. The e-nose model achieved an accuracy of 87% for rancidity classification, while prediction models based on e-nose data showed a strong relationship with the GC-MS aroma measurements. This study provides a strong example of how low-cost and potentially portable sensor systems can be used to assess oxidative defects in edible oils [171].

The combination of sensor data and conventional reference methods is particularly important for oil applications. Unlike simple classification approaches, studies such as those described above relate changes in the volatile fingerprint to measurable chemical deterioration. Parameters including malondialdehyde, p-anisidine value, oxygen induction time, total polar compounds, and GC-MS volatile profiles provide complementary information that can help establish the relationship between sensor responses and the progression of oxidation.

Overall, recent studies demonstrate that e-noses can provide rapid information about oxidative deterioration and rancidity in edible oils, particularly when combined with chemometric or machine learning approaches. Edible oils therefore represent one of the strongest application areas for mini electronic noses because lipid oxidation is directly reflected in the evolution of the headspace composition. Future systems should focus on continuous or repeated monitoring during storage and frying and should integrate sensor responses with reference markers such as peroxide value, p-anisidine value, total polar compounds, TBARS where appropriate, and targeted analysis of oxidation-derived aldehydes and ketones [170].

#### 4.6.5. Other Lipid-Containing Food Matrices

Beyond meat, dairy products, fermented foods, and edible oils, other lipid-containing food matrices represent promising but still relatively underexplored applications for miniaturized electronic noses. Electronic-nose technologies have already been widely investigated for food-quality assessment, freshness evaluation, authentication, adulteration detection, and general sample classification. However, many of these studies focus on discriminating samples according to product type, origin, storage condition, or overall quality without directly linking sensor responses to chemically validated lipid oxidation markers. Consequently, although sensor arrays can successfully detect changes in complex volatile fingerprints, their application to the specific monitoring of oxidative deterioration requires a more targeted experimental and analytical approach [79].

Complex foods containing unsaturated lipids, such as plant-based products, emulsified foods, sauces, bakery products, and other formulated foods, may represent particularly interesting targets for future applications. In these matrices, lipid oxidation can contribute to shelf-life reduction and sensory deterioration, but the interpretation of volatile profiles is more challenging than in relatively simple matrices such as edible oils. Indeed, VOCs detected in the headspace may originate simultaneously from lipid oxidation, microbial activity, protein degradation, thermal processing, or other matrix-specific reactions. The broad cross-sensitivity of electronic-nose sensor arrays can therefore make it difficult to attribute a specific sensor response exclusively to lipid oxidation, particularly when multiple sources of volatile compounds coexist within the same food matrix [172].

For these complex matrices, the integration of mini electronic noses with complementary analytical techniques could substantially improve the interpretation of sensor responses. While an electronic nose provides a rapid global fingerprint of the volatile headspace, chromatographic techniques such as GC–MS can identify specific compounds responsible for changes in the volatile profile. Combining these approaches can therefore help establish relationships between sensor signals and relevant chemical markers, including oxidation-derived aldehydes, ketones, alcohols, and other VOCs. Such integrated strategies are particularly important when the objective is not simply to classify samples but to understand the chemical processes underlying the observed sensor responses [107].

Future studies should therefore move beyond general food classification and validate mini e-nose responses against specific markers of lipid oxidation. Depending on the food matrix, these markers may include peroxide value, lipid hydroperoxides, p-anisidine value, malondialdehyde or TBARS, as well as targeted volatile compounds such as pentanal, hexanal, heptanal, octanal, and nonanal. Establishing direct correlations between these conventional reference measurements and sensor features would facilitate the identification of sensor-response patterns specifically associated with oxidative deterioration. The integration of chemometric and machine learning approaches may further support the development of predictive models capable of estimating oxidation status rather than simply separating samples into predefined categories [79].

Overall, other lipid-containing food products constitute an emerging application area for mini electronic noses. Current research demonstrates the ability of portable and low-cost sensor systems to analyse complex food headspaces, but challenges remain regarding the chemical specificity of sensor responses, sensor drift, and the reliability of models under real-world conditions. Future research should therefore focus on establishing reproducible relationships between sensor fingerprints, specific oxidation-derived VOCs, conventional lipid oxidation parameters, and sensory perception of rancidity. Such an approach could extend the use of mini electronic noses from general food classification towards more reliable, rapid, and potentially real-time monitoring of oxidative shelf life in a wider range of lipid-containing food products [7].

### 4.7. Data Analyses

The interpretation of e-nose data relies on chemometric and machine learning approaches, as raw sensor signals are inherently multivariate and not directly interpretable [84]. Figure 5 summarises the typical workflow for mini e-nose data processing in food-quality assessment applications. Commonly applied techniques include principal component analysis (PCA), linear discriminant analysis (LDA) [173], support vector machines (SVMs) and artificial neural networks (ANNs), which are employed for dimensionality reduction, feature extraction, and classification of VOC-based patterns [174]. These methods convert complex sensor responses into either reduced-dimensional visualizations or predictive models that capture differences in VOC profiles.

Convolutional neural networks (CNNs) have been successfully used to extract spatial features from multi-channel sensor arrays and to improve odour classification and food-quality prediction. Recurrent neural networks (RNNs)—in particular, architectures with long short-term memory (LSTM) and gated recurrent units (GRUs)—are particularly suitable for modelling the temporal dynamics of sensor responses during gas adsorption and desorption cycles. More recently, transformer-based architectures have emerged as promising alternatives because their self-driving mechanism attention can capture long-range dependencies within multivariate sensor signals while allowing for parallel processing and improved scalability. Attention mechanisms further improve the interpretability of models by identifying the most informative sensors or time intervals contributing to odour discrimination. These deep learning approaches have demonstrated superior performance over conventional machine learning algorithms for odour classification, gas concentration prediction, the compensation of sensor drift and the complex evaluation of food quality [175].

To facilitate the comparison of these approaches, Table 4 summarizes the main chemometric and machine learning algorithms used in electronic-nose applications, highlighting their principal strengths, limitations, and relevance to miniaturized e-nose systems.

As reported in the literature, the e-nose outputs typically constitute multivariate datasets that require statistical and computational modelling for meaningful interpretation rather than direct chemical identification [178]. This reliance on chemometrics is a fundamental aspect of the technology and distinguishes e-noses from traditional analytical instruments, which typically provide direct compound-level measurements [179]. Among these methods, PCA is one of the most widely used unsupervised chemometric tools because it enables visualization of similarities and differences between samples by reducing high-dimensional sensor data into a small number of principal components while preserving the maximum variance. PCA score plots are frequently used to discriminate food products according to freshness, geographical origin, adulteration, fermentation stage, or spoilage level. In contrast, supervised techniques such as LDA maximize the separation between predefined sample groups and are therefore more suitable for classification purposes [180]. These methods are particularly useful in food authentication studies where e-nose systems must distinguish between products with subtle aromatic differences. As can be seen in Figure 5, before chemometric analysis, e-nose signals generally undergo several preprocessing steps to improve model reliability and reduce sensor-drift effects. Common preprocessing techniques include baseline correction, normalization, smoothing, outlier removal, and drift compensation [175]. Sensor drift is considered one of the major limitations of MOS sensor-based e-noses because sensor sensitivity may gradually change over time due to aging, humidity, or temperature variations. Consequently, calibration transfer methods and adaptive machine learning algorithms are often required to maintain long-term prediction accuracy. As reported in the literature, e-nose outputs generally constitute multivariate datasets that require statistical and computational modelling for meaningful interpretation rather than direct chemical identification. This reliance on chemometrics fundamentally distinguishes e-noses from traditional analytical techniques such as GC-MS, which provide direct compound-level identification and quantification. Instead of identifying individual VOCs, e-noses recognize global aroma fingerprints, mimicking the functioning principles of the human olfactory system [107]. Consequently, the performance of an e-nose depends not only on sensor quality but also on the efficiency of the chemometric algorithms used for pattern recognition and predictive analysis [181].

The rapid development of artificial intelligence (AI) and deep learning (DL) has substantially expanded the analytical capabilities of e-nose systems. Recent developments have increasingly focused on convolutional neural networks (CNNs), which can automatically extract discriminative features from raw or minimally processed sensor responses [182]. Unlike traditional approaches requiring manual feature extraction, CNN-based models can learn temporal or spatial patterns directly from sensor signals. Another major recent development is the use of deep learning to address sensor drift, which remains one of the main obstacles to the long-term deployment of e-nose systems. Sensor responses can change over time because of sensor aging, environmental variations, humidity, temperature, and changes in operating conditions. Conventional drift compensation approaches often require substantial recalibration or labelled data from the target measurement period. Recent studies have therefore explored deep learning-based domain adaptation and representation-learning strategies. A 2024 study introduced a masked autoencoder module for sensor-drift compensation, demonstrating the potential of self-supervised representation learning to extract stable features from drifting sensor responses [183]. Similarly, a semi-supervised adversarial domain-adaptive CNN was proposed in 2025 to compensate for long-term drift and sensor displacement, using a one-dimensional CNN as a feature extractor while reducing discrepancies between source and target domains [184]. These approaches are particularly promising for mini e-noses because they could reduce the frequency of manual recalibration and improve the stability of portable devices during repeated or long-term measurements.

PCA and LDA remain particularly useful for mini e-noses because of their relatively low computational requirements and their suitability for implementation on devices with limited processing resources. PCA is primarily an unsupervised dimensionality-reduction method and is therefore especially valuable for visualizing sensor patterns, identifying clusters, detecting outliers, and reducing redundancy before subsequent modelling. However, PCA does not directly optimize class separation and should not be interpreted as a classification model. LDA, by contrast, is supervised and explicitly maximizes separation between predefined classes. It is therefore useful when the objective is rapid discrimination between a limited number of oxidation states or food-quality categories. Both methods are generally more interpretable than complex neural networks and are attractive for portable devices, although LDA may become unstable when the number of variables is high relative to the number of samples or when its statistical assumptions are not adequately satisfied [84].

PLS-DA occupies an intermediate position between exploratory chemometrics and machine learning. Its principal advantage is that it simultaneously reduces dimensionality and constructs latent variables associated with class discrimination, which is particularly relevant for correlated e-nose signals. Compared with black-box models, PLS-DA can provide greater interpretability through variable contributions and the relationship between sensor responses and sample classes. This can be valuable when the objective is not only to classify fresh and oxidized samples but also to identify which sensors or signal features contribute most strongly to discrimination. However, PLS-DA can easily produce overly optimistic results if the number of latent variables is not carefully optimized or if model validation is inadequate. Cross-validation and, preferably, external validation should therefore be used to control overfitting [185].

SVM is often particularly suitable for e-nose datasets because sensor systems can generate numerous correlated features while the number of available samples remains relatively limited. By constructing a decision boundary with maximum separation between classes, SVM can perform well in high-dimensional spaces without necessarily requiring the very large datasets typically associated with deep learning. Non-linear kernels can further improve discrimination when oxidation-related sensor patterns are not linearly separable. However, model performance depends strongly on hyperparameter selection and kernel choice. Excessively flexible kernels or poorly optimized parameters may lead to overfitting, particularly when validation datasets are small. Although the computational cost of model training can increase with dataset size, the inference stage is generally feasible for portable systems when the final model remains sufficiently compact [186].

Random forest can model non-linear relationships and interactions between sensor features without requiring the extensive data preprocessing often needed for linear methods. Its ensemble structure also makes it relatively resistant to overfitting compared with a single decision tree, particularly when an appropriate number of trees and validation procedures are used. Another advantage is the possibility of estimating variable importance, which can help identify the sensors or extracted features contributing most strongly to classification. Nevertheless, increasing the number and depth of trees increases memory use and computational cost. In addition, although variable importance improves interpretability, the complete model remains less transparent than PCA, LDA, or a simple PLS-DA model. Random forest is therefore attractive for moderately sized datasets and more powerful portable processors but may be less appropriate for highly constrained embedded devices [187].

ANNs become advantageous when the relationship between sensor signals and food quality is highly non-linear and cannot be adequately represented by conventional chemometric models. They can learn complex interactions between multiple sensors and extracted temporal features, potentially improving classification or prediction performance. However, ANN performance is strongly dependent on architecture selection and dataset size. When the number of trainable parameters is large relative to the number of independent samples, the risk of overfitting increases substantially. ANNs are also less interpretable because it is difficult to directly relate a classification decision to a specific sensor response. Their training is computationally more demanding than PCA, LDA, or PLS-DA, although inference using a small, optimized network may still be feasible on portable hardware. Consequently, ANN models should be considered when simpler models fail to capture the relevant non-linear relationships and when sufficient independent training data are available [185].

Deep learning approaches can be particularly valuable when raw or time-resolved sensor signals contain complex temporal patterns that would otherwise require manual feature extraction. They may therefore improve automated feature learning and classification accuracy. However, this potential must be balanced against their substantially greater requirements for training data and computational resources. Small experimental datasets are especially problematic because a deep model with many parameters can memorize the training data rather than learn reproducible oxidation-related patterns. Recent e-nose reviews continue to identify limited generalizability, weak external validation, and small datasets as major barriers to real-world deployment. Deep learning may therefore be more appropriate for large datasets collected across multiple batches, devices, storage conditions, and food matrices. For portable systems, deployment may require model compression, quantization, or lightweight architectures for edge computing [188].

Overall, no single algorithm can be considered universally optimal for mini e-noses. The selection of data-analysis method should depend on the dataset size, feature dimensionality, required interpretability, available computational resources, and final deployment scenario. PCA and LDA are particularly attractive for exploratory analysis and low-resource applications, whereas PLS-DA provides a useful compromise between discrimination and chemical interpretability. SVM is often well suited to relatively small and high-dimensional e-nose datasets, while random forest can capture non-linear relationships and provide feature-importance information at the expense of increased model complexity. ANN and deep learning approaches offer greater capacity to model complex sensor responses but require more extensive datasets and careful validation to avoid overfitting. Therefore, for portable mini e-nose systems intended for food-quality monitoring, simpler models should generally be considered first, and more complex algorithms should only be adopted when they provide a reproducible improvement confirmed by independent external validation [75].

As illustrated in Figure 5, the analytical workflow begins with sample measurement, during which the mini e-nose is exposed to free space above the food sample. The preprocessing and feature extraction step is essential to improve data quality before model development. Typical preprocessing procedures include basic correction, normalization, smoothing, filtering, drift compensation and noise reduction. After the extraction of features, chemometric and machine learning methods are applied to identify patterns and establish predictive models. Exploratory techniques such as PCA are commonly used to visualize data structure and detect groupings or outliers, while supervised algorithms, including ADL, SVM, random forest (RF), K-nearest neighbours (KNN), artificial neural networks (ANNs), and, more recently, convolutional neural networks (CNNs), long short-term memory (LSTM) networks and transformer-based models, are used for classification and quantitative prediction. These approaches allow for the recognition of complex scent fingerprints while improving robustness to sensor variability and environmental fluctuations [175].

## 5. Interpretation, Limitations, and Practical Implications

### 5.1. Interpretation of E-Nose Responses in Food-Quality Monitoring

The analysis of the literature presented in this review shows that e-noses are a particularly promising technology for rapid, non-destructive and decentralized control of food quality. Their main interest, however, lies not in their ability to replace conventional analytical methods, but in their ability to quickly generate a global footprint of the volatile profile of a food. This characteristic is particularly interesting in situations where many samples must be analysed quickly or when the inspection must be carried out directly at the place of production, storage or distribution. The development of miniaturized systems further reinforces this interest by reducing the size, cost and complexity of devices. This article shows that the evolution of e-nose responses can be associated with different levels of degradation in oils, meat products, dairy products and other food matrices. However, this relationship must be interpreted with caution. The presence of a change in the signal of an e-nose is not in itself direct evidence of increased lipid oxidation. The sensors detect a complex mixture of volatile compounds and not a specific molecule. An evolution in their response can therefore result from several simultaneous phenomena, including lipid oxidation, but also other chemical or biochemical reactions specific to the matrix studied. The role of the e-nose is thus more that of a profile recognition system than that of an instrument for direct molecular analysis. This distinction is an essential point for interpreting the performances reported in the literature. Excellent classification capability means that the system can differentiate volatile fingerprints from several sample groups but does not necessarily mean that sensors identify which molecules are responsible for this separation. The power of an e-nose comes precisely from the collective response of the network: each sensor has a partial selectivity and responds to several compounds, while the combination of responses generates a sufficiently reproducible footprint to differentiate complex mixtures.

### 5.2. Comparison of Sensing Technologies

The performance of mini e-noses cannot be attributed to a single “best” sensor technology, as the suitability of a sensing platform depends on the physicochemical properties of the target VOCs, the required detection limit, the operating environment, and the intended monitoring strategy. Among the available platforms, metal oxide semiconductor (MOS) sensors remain the most widely used technologies because they combine high sensitivity, low cost, rapid response, and straightforward miniaturization. Their performance is particularly advantageous for applications requiring repeated or continuous monitoring of oxidation-related VOCs, such as aldehydes and ketones generated during lipid oxidation. However, their broad cross-sensitivity and susceptibility to humidity, temperature variations, and long-term sensor drift can limit chemical specificity and reproducibility. MOS-based systems are therefore particularly suitable when the objective is to detect changes in a global volatile fingerprint rather than to selectively quantify a single oxidation compound [189].

Other sensing platforms may provide advantages when greater chemical selectivity is required. Electrochemical sensors can offer selective detection of specific gases or reactive compounds and can operate at relatively low power, making them potentially attractive for portable systems. However, their lifetime may be limited, and their performance can be affected by cross-interference, electrolyte degradation, and environmental conditions. Consequently, electrochemical sensing may be more appropriate when a limited number of well-defined target compounds are known, whereas MOS arrays are generally more suitable for broad VOC fingerprinting [118].

Quartz crystal microbalance (QCM) and other mass-sensitive sensors can provide high sensitivity because VOC adsorption produces measurable changes in mass or resonance frequency. Their performance strongly depends on the choice of coating or functional layer, which can improve selectivity toward chemical classes. These platforms may therefore be advantageous when the monitoring objective is focused on specific oxidation-related VOCs. Nevertheless, their sensitivity to temperature, humidity, mechanical disturbances, and coating aging can complicate their use outside controlled laboratory conditions [132].

Optical and photonic sensing platforms can provide high sensitivity and, depending on the recognition material, improved selectivity and chemical discrimination. They are promising for miniaturized and multiplexed devices, particularly when combined with machine learning algorithms. However, fabrication complexity, material stability, optical alignment, and cost can currently limit large-scale implementation compared with conventional MOS sensors [190].

From an application perspective, sensor selection should therefore be based on the analytical objective rather than on sensitivity alone. For low-cost, portable, or embedded monitoring of general oxidation trends, MOS-based arrays currently offer the most practical compromise between sensitivity, size, power consumption, and cost. When improved selectivity towards specific VOCs is required, QCM, optical, or functionalized sensor platforms may be more appropriate. Electrochemical sensors may be advantageous for targeted detection of a limited number of analytes, whereas more sophisticated analytical systems, such as fast GC-based electronic noses, provide greater chemical discrimination but sacrifice simplicity, miniaturization, and suitability for continuous monitoring [84].

### 5.3. Challenges for Industrial Implementation

Despite significant technological progress, several barriers continue to limit industrial implementation. Sensor drift and aging remain major challenges for long-term operation, while humidity and temperature fluctuations can substantially modify sensor responses. In addition, the transferability of calibration models between devices, food matrices, production sites, and storage conditions remains limited. Industrial deployment will therefore require robust calibration-transfer procedures, automated drift compensation, standardized sampling protocols, and long-term validation under realistic processing and storage conditions. The development of hybrid systems combining complementary sensor technologies may further improve reliability, but this approach must be balanced against increased cost, complexity, and power consumption [191].

One important consideration is the total cost of ownership rather than the initial price of the electronic nose alone. Miniaturized systems based on commercially available MOS sensors and integrated microcontrollers can potentially reduce hardware costs and facilitate large-scale deployment. However, the total cost must also include sensor replacement, calibration procedures, maintenance, data-processing infrastructure, and potential replacement of components affected by contamination or long-term use. Thus, the economic feasibility of e-nose systems should be evaluated based on their complete operational costs rather than hardware acquisition costs alone. 

Calibration and sensor drift represent major challenges for long-term deployment. Most laboratory studies calibrate sensors before experiments and evaluate model performance over relatively short periods. In industrial applications, however, sensor responses may change over weeks or months because of aging, poisoning, contamination, temperature fluctuations, and humidity variations, and exposure to complex VOCs mixtures. In food-processing environments, exposure to aerosols, fats, cleaning agents, and varying humidity may further affect sensor performance. Consequently, periodic recalibration and drift-correction procedures, and sensor replacement may be required, with replacement intervals depending strongly on the food matrix and operationg environment rather than on a fixed manufacturer-defined lifetime. The appropriate calibration frequency cannot currently be generalized because it depends on sensor technology, operating conditions, exposure intensity, and the required analytical accuracy. Future studies should therefore report calibration intervals, baseline stability, sensor replacement frequency, and model performance over extended periods rather than only reporting initial classification accuracy [192].

### 5.4. Standardization and Reproducibility

Another major barrier is the lack of standardized protocols for mini electronic-nose measurements. Differences in sampling volume, headspace equilibration time, temperature, humidity, carrier gas or airflow, sensor operating conditions, signal preprocessing, feature extraction, and machine learning validation can make direct comparison between studies difficult. Standardized operating procedures would be particularly important for industrial applications because calibration models developed using one device or production site may not necessarily be directly transferable to another. Future work should therefore focus on common protocols for sampling, calibration, preprocessing, model validation, and performance reporting.

### 5.5. Regulatory Considerations and Analytical Validation

Regulatory acceptance represents an additional challenge. E-noses are generally more likely to be adopted initially as rapid screening or process-monitoring tools rather than as direct replacements for established reference methods. For critical food-quality or safety decisions, e-nose measurements may need to be validated against recognized analytical methods, such as GC–MS, peroxide value, p-anisidine value, TBARS, or microbiological methods, depending on the application. The development of validated performance criteria, including accuracy, precision, repeatability, reproducibility, robustness, and limits of detection, will therefore be necessary before broader regulatory acceptance can be achieved.

One important consideration is the total cost of ownership rather than the initial price of the electronic nose alone. Miniaturized systems based on commercially available MOS sensors and integrated microcontrollers can potentially reduce hardware costs and facilitate large-scale deployment. However, the total cost must also include sensor replacement, calibration procedures, maintenance, data-processing infrastructure, and potential replacement of components affected by contamination or long-term drift. In food-processing environments, sensors may be exposed to humidity, aerosols, fats, cleaning agents, and complex VOC mixtures, which can progressively alter sensor responses. Consequently, sensor replacement intervals may depend strongly on the food matrix and operating environment rather than on a fixed manufacturer-defined lifetime [193].

Calibration and sensor drift represent major challenges for long-term deployment. Most laboratory studies calibrate sensors before experiments and evaluate model performance over relatively short periods. In industrial applications, however, sensor responses may change over weeks or months because of aging, poisoning, contamination, temperature fluctuations, and humidity variations. Periodic recalibration and drift-correction procedures are therefore likely to be required. The appropriate calibration frequency cannot currently be generalized because it depends on sensor technology, operating conditions, exposure intensity, and the required analytical accuracy. Future studies should therefore report calibration intervals, baseline stability, sensor replacement frequency, and model performance over extended periods rather than only reporting initial classification accuracy [97].

Another major barrier is the lack of standardized protocols for mini e-nose measurements. Differences in sampling volume, headspace equilibration time, temperature, humidity, carrier gas or airflow, sensor operating conditions, signal preprocessing, feature extraction, and machine learning validation can make direct comparison between studies difficult. Standardized operating procedures would be particularly important for industrial applications because calibration models developed using one device or production site may not necessarily be directly transferable to another. Future work should therefore focus on common protocols for sampling, calibration, preprocessing, model validation, and performance reporting [194]. Regulatory acceptance represents an additional challenge. Electronic noses are generally more likely to be adopted initially as rapid screening or process-monitoring tools rather than as direct replacements for established reference methods. For critical food-quality or safety decisions, e-nose measurements may need to be validated against recognized analytical methods, such as GC–MS, peroxide value, p-anisidine value, TBARS, or microbiological methods, depending on the application. The development of validated performance criteria, including accuracy, precision, repeatability, reproducibility, robustness, and limits of detection, will therefore be necessary before broader regulatory acceptance can be achieved [195].

Overall, industrial implementation should not be evaluated solely according to classification accuracy. A practical industrial mini e-nose must maintain reproducible performance despite sensor drift, environmental variability, contamination, and changes in raw materials or production batches. Future studies should therefore include long-term validation under realistic industrial conditions and report practical parameters such as sensor lifetime, replacement frequency, recalibration intervals, maintenance requirements, computational resources, and total operational cost. Addressing these factors will be essential for determining whether mini electronic noses can move beyond laboratory demonstrations toward reliable large-scale food-quality monitoring [196].

One of the main lessons from this review is that the performance of an e-nose depends as much on data processing as on the sensors themselves. Raw signals obtained from a sensor array are multidimensional and generally do not allow for direct interpretation. Methods such as PCA, LDA, SVM, ANN or other machine learning approaches are therefore needed to transform sensor responses into usable information.

However, **sensor drift** is probably one of the main obstacles to the long-term use of e-noses. The response properties of sensors can change with aging, temperature, humidity or repeated exposure to certain compounds. Therefore, a chemometric model calibrated at a given time can gradually lose its predictive capacity when the characteristics of the sensors change [184]. This issue is particularly critical for a system intended for real-time monitoring of lipid oxidation. A variation in the signal over time can be interpreted as a change in the volatile profile, whereas it may result from a change in the sensor’s behaviour. Using an e-nose to monitor the evolution of an oil over several weeks or months therefore requires a specific drift control strategy [175]. Another important source of variability is relative humidity. Water molecules can interact with the surface of sensors and modify the available sites for adsorption of VOCs. This interaction can modify the sensitivity and basic resistance of the sensors, resulting in a variation in the signal that is not necessarily related to the change in product composition [191]. Temperature, air flow and sampling conditions are also factors that may affect responses. This limitation is particularly important for food applications, as samples can be analysed under different conditions depending on their storage temperature or environment.

## 6. Future Direction

Future research should first aim at improving the stability of mini e-noses under real conditions. The integration of temperature and humidity sensors, combined with environmental compensation algorithms, could reduce non-VOC variations. Drift correction and automatic recalibration strategies should also be developed to allow prolonged use without frequent manual recalibration. Sensor development should additionally focus on improving sensitivity, selectivity, response time, and long-term stability. A second important direction concerns the development of hybrid systems. The combination of a mini-e-nose with another rapid technique such as NIR spectroscopy could combine information on volatile compounds with that related to the overall composition of the oil. Results obtained for sunflower, rapeseed, and flaxseed oils already suggest that such data fusion can provide more comprehensive information than e-nose measurements alone [197]. A third priority would be to develop models specifically designed to monitor lipid oxidation. Future studies should simultaneously measure peroxide value for primary oxidation products, TBARS for secondary oxidation products, and GC-MS for specific VOCs. Combining these indicators would allow a more robust relationship to be established between the mini e-nose fingerprint and the different stages of oxidation. At the same time, improved machine learning and deep learning approaches should be developed to account for sensor drift, environmental variability, and differences between samples and devices. Finally, future research should focus on industrial translation and regulatory standardization. Mini-e-noses should be validated under realistic processing and storage conditions and across different batches and devices. Standardized protocols for calibration, reproducibility, environmental robustness, and validation against reference methods such as TBARS and GC-MS will be essential to support regulatory acceptance and facilitate the adoption of mini e-noses as rapid, complementary tools for real-time lipid oxidation monitoring. Future research should also explore the integration of mini e-noses with complementary sensing technologies, such as electronic tongues (e-tongues), electronic eyes (e-eyes), and other electrochemical sensors, to enable multimodal food-quality assessment with improved accuracy and robustness.

## 7. Conclusions

All the data presented in this review show that mini e-noses represent a particularly promising technology for fast and non-destructive monitoring of food quality. Their main interest is not in their ability to replace analytical reference techniques, but in their ability to quickly provide a global footprint of volatile compounds and detect changes associated with product degradation. This feature makes them particularly interesting for applications requiring frequent, decentralized or near real-time monitoring. In the field of lipid oxidation, this technology is particularly relevant since the degradation of lipids generates a complex succession of primary and secondary products, including several volatile compounds responsible for rancidity odours. Studies on vegetable oils show that the signatures obtained with e-noses evolve with the degree of degradation and can be exploited by chemometric methods to differentiate different oxidation states. However, performances reported in the literature should be interpreted with caution. Excellent classification or high statistical correlation does not necessarily mean that an e-nose directly measures lipid oxidation. Sensors are sensitive to complex mixtures of VOCs, and their response can be influenced by temperature, humidity, matrix, sensor aging and sampling conditions. The e-nose should therefore be considered a pattern recognition system rather than a molecular analytical instrument. This distinction is probably one of the main points to reinforce in the current literature.

## Figures and Tables

**Figure 3:**
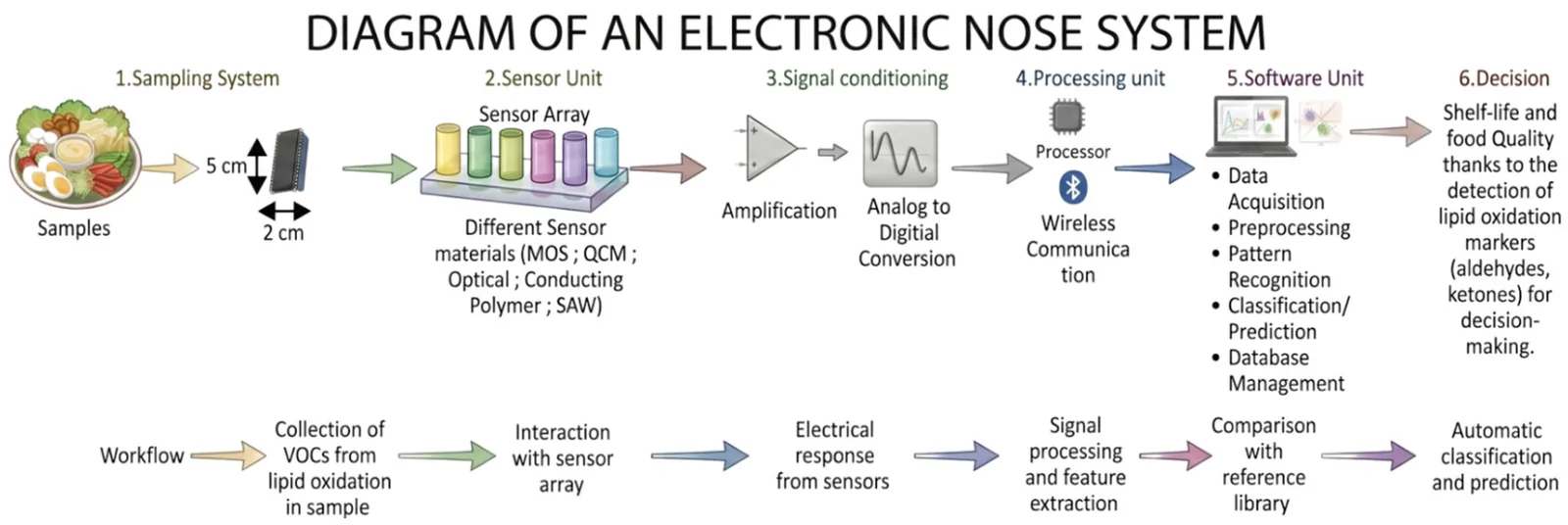
Diagram of the e-nose system created in BioRender (https://www.biorender.com/). As shown in this figure, an electronic nose consists of several interconnected modules that transform VOCs emitted from a food sample into digital information for quality assessment. The process begins with sampling the headspace, where the VOCs are transported to a sensor network composed of partially selective sensors The interaction between the VOC molecules and the sensor surfaces generates characteristic electrical responses that are then amplified, digitized, and transmitted to the processing unit. The signal preprocessing, including base correction, normalization, filtering and drift compensation, is then applied before feature extraction and pattern recognition.

**Figure 4:**
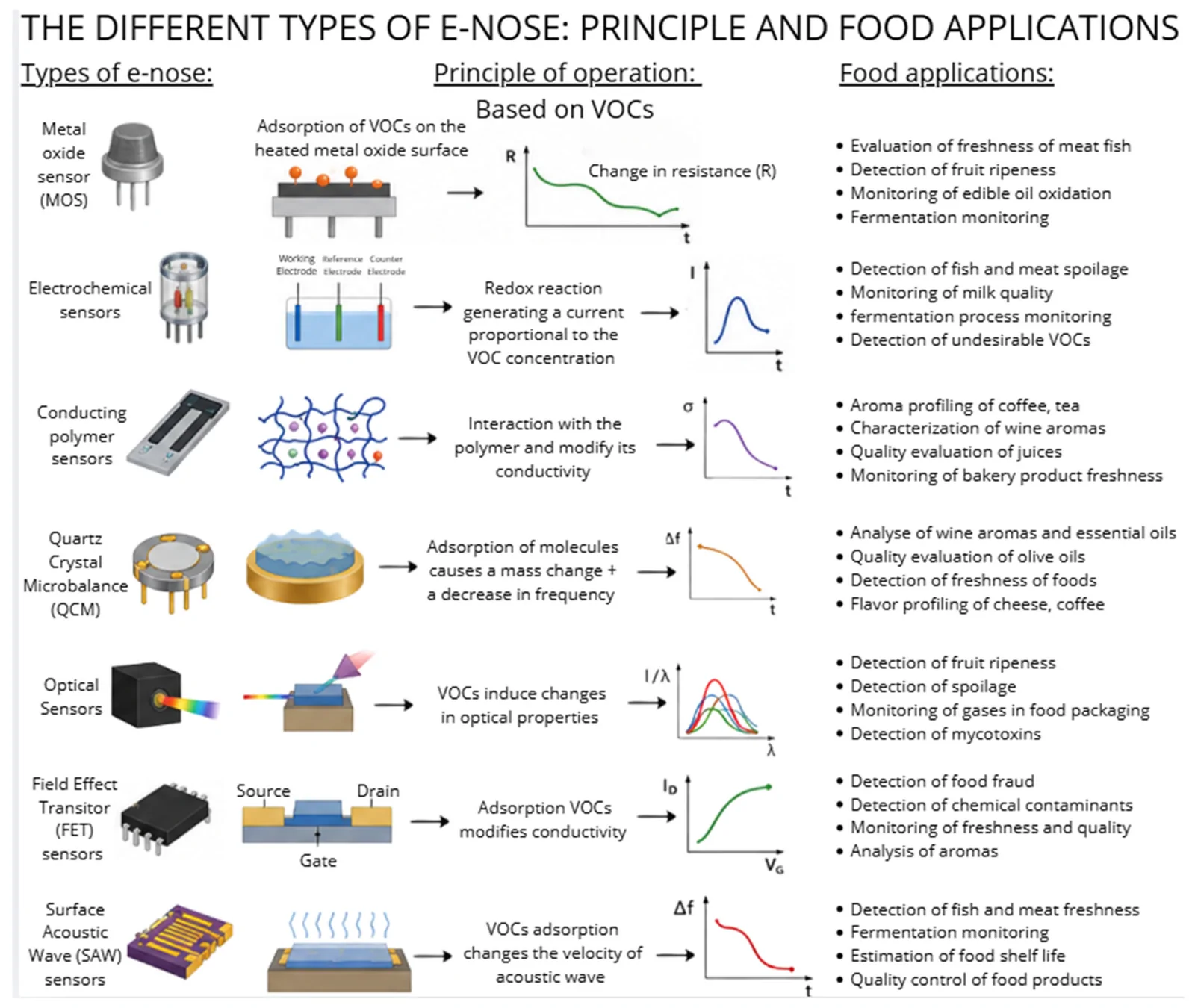
Main sensor technologies used in e-nose systems. Created in BioRender (https://www.biorender.com/).

**Figure 5:**
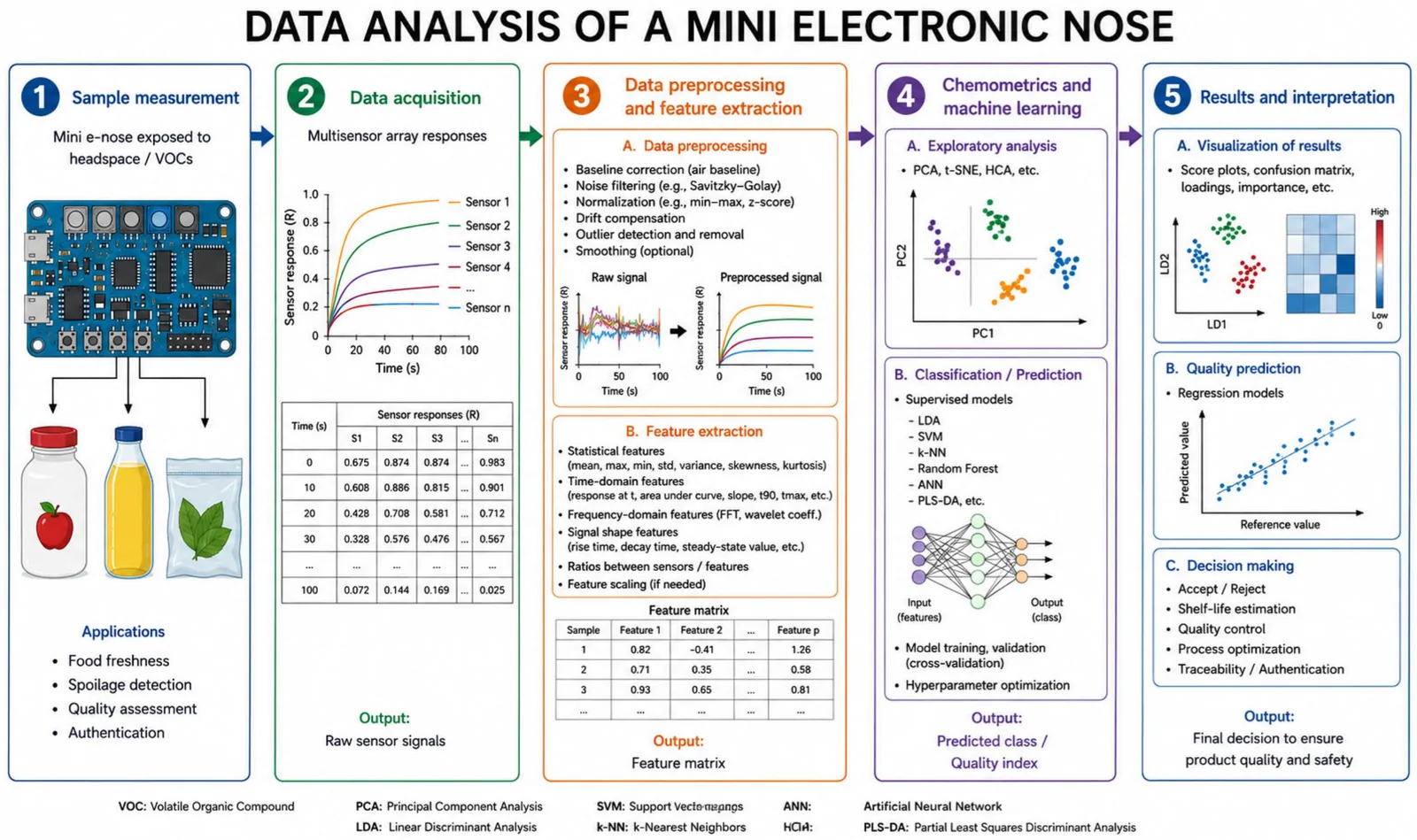
Data analysis of a mini electronic nose created in BioRender (https://www.biorender.com/).

**Table 1:** Major volatile and non-volatile compounds generated during lipid oxidation and their relevance for food-quality monitoring and e-nose applications.

Compound Class	Representative Compounds	Origin During Lipid Oxidation	Quality Relevance	Relevance for E-Nose Monitoring	References
Aldehydes	Hexanal, pentanal, nonanal	Hydroperoxide decomposition	Rancidity and off-flavour formation; important oxidation indicators	Major VOC markers responsible for changes in sensor response patterns	[1]
Ketones	2-Heptanone, 2-nonanone, 2,4-heptadienone	Secondary oxidation reactions	Development of undesirable flavours	Contribute to volatile fingerprints detected by sensor arrays	[4]
Alcohols	1-Pentanol, 1-hexanol, 1-octen-3-ol	Reduction of aldehydes and lipid oxidation pathways	Changes in aroma characteristics	Influence overall VOC profiles detected by e-noses	[2]
Hydrocarbons	Pentane, hexane, ethane	Fatty acid degradation pathways	Indicators of oxidative degradation	Contribute to oxidation-related VOC patterns	[3]
MDA	Malondialdehyde	Hydroperoxide decomposition	Quantitative marker measured by TBARS	Used as reference parameter for validating e-nose predictions	[1]

**Table 3:** Comparison of commercially available electronic-nose systems for food-quality monitoring and their suitability for industrial applications.

Category	Company	Commercial System	Sensor/Detection Technology	Main Advantages	Food Applications	Practical Limitations	Industrial Suitability
**Miniature**	Stratuscent	E-nose MX	Miniaturized VOC sensors	Low-cost, small size, IoT integration potential	Continuous VOC monitoring, food freshness tracking	Selectivity, calibration transfer, long-term stability challenges	Promising for future connected food-monitoring systems
**Miniature**	Bosch Sensortec	BME688/BME690	Semiconductor VOC sensors	Small size, low power consumption, integration with electronic platforms	Dairy products, oils, beverages, fermented products, smart packaging	Limited selectivity, requires machine learning calibration	
**Miniature**	Aryballe	NeOse Pro/Sense Kit	Biomimetic chemical sensing + machine learning	Compact design, digital aroma recognition, portable platform	Aroma fingerprinting, food and beverage-quality assessment, authentication	Requires further validation across different matrices and environments	Promising for portable applications; industrial deployment still developing
**Portable**	Sensigent	eNose MSEM 1400/Cyranose 320/eNose QA	Sensor-array-based VOC detection	Flexible configurations, portable, field applications	Contamination detection, off-flavour detection, quality assurance	Sensor stability, calibration requirements, limited long-term validation	Suitable for screening applications; long-term industrial use requires improvement
**Portable**	Electronic Sensor Technology	zNose 4300/4650	Fast GC-based VOC analysis	High chemical specificity and compound identification capability	Flavour analysis, beverage monitoring, contamination detection	Higher cost, larger instrumentation, limited portability compared with sensor-array systems	Suitable for laboratory-based industrial quality assessment
**Portable**	Tellspec	Tellspec Sensor/Preemie^TM^	Spectroscopic sensing	Compact design, rapid analysis potential	Freshness and contamination assessment of packaged foods	Requires further validation and calibration for complex food matrices	Potential for smart packaging and consumer applications
**Miniature**	Stratuscent	E-nose MX	Miniaturized VOC sensors	Low cost, small size, IoT integration potential	Continuous VOC monitoring, food freshness tracking	Selectivity, calibration transfer, long-term stability challenges	Promising for future connected food-monitoring systems
**Miniature**	Alpha MOS	FOX 4000/FOX 6000/HERACLES Neo	Sensor arrays; ultra-fast GC-based system (HERACLES Neo)	High analytical capability, advanced data analysis, good reproducibility	Wine classification, dairy and meat-quality assessment, beverage analysis, authentication	Higher cost, complex operation, less suitable for low-cost continuous monitoring	Suitable for quality control laboratories and advanced industrial analysis

**Table 4:** Comparative strengths, limitations, and applications of chemometric and machine learning algorithms in mini e-nose systems for food-quality monitoring.

Algorithm	Advantage	Limitation	Relevance to Mini E-Nose	References
**PCA**	Simple, interpretable dimensionality reduction	Unsupervised; not predictive	High for exploratory analysis	[176]
**LDA**	Simple and efficient classification	Distribution/covariance assumptions	High for small datasets	[173]
**PLS-DA**	Handles collinearity and high-dimensional data	Overfitting risk	High	[176]
**PLSR**	Predicts continuous reference values	Mainly linear	Very high for TBARS correlation	[176]
**SVM**	Strong nonlinear classification	Hyperparameter optimization required	Very high for small datasets	[174]
**Random forest**	Nonlinear, robust, feature importance	Less interpretable; overfitting risk	High	[177]
**ANN**	Models complex nonlinear relationships	Data-hungry; overfitting	Moderate–high	[174]
Deep learning (CNN/DNN)	Learning complex patterns automatically from large datasets; reduces need for manual feature extraction	Requires large datasets; high computational demand; limited interpretability	Increasing potential for advanced VOC pattern recognition and multi-sensor data fusion	[78]

## Data Availability

No new data were created or analyzed in this study. Data sharing is not applicable to this article.
